# The cytotoxic activity of carfilzomib together with nelfinavir is superior to the bortezomib/nelfinavir combination in non-small cell lung carcinoma

**DOI:** 10.1038/s41598-023-31400-6

**Published:** 2023-03-17

**Authors:** Lenka Besse, Marianne Kraus, Andrej Besse, Christoph Driessen, Ignazio Tarantino

**Affiliations:** 1 Laboratory of Experimental Oncology, Department of Oncology and Hematology, Cantonal Hospital St. Gallen, 9000 St. Gallen, Switzerland; 2 Department of General, Visceral, Endocrine and Transplant Surgery, Kantonsspital St. Gallen, 9000 St. Gallen, Switzerland; 3 Cantonal Hospital St. Gallen, Rorschacherstrasse 95 Haus 09/218, 9007 St. Gallen, Switzerland

**Keywords:** Non-small-cell lung cancer, Preclinical research, Chemotherapy, Cancer therapeutic resistance, Chemical tools

## Abstract

Chemotherapy resistance is still a major problem in the treatment of patients with non-small-cell-lung carcinoma (NSCLC), and novel concepts for the induction of cytotoxicity in NSCLC are highly warranted. Proteotoxicity, the induction of cytotoxicity by targeting the ubiquitin proteasome system, represents an appealing innovative strategy**.** The combination of the proteasome inhibitor bortezomib (BTZ) and the proteotoxic stress-inducing HIV drug nelfinavir (NFV) synergistically induces proteotoxicity and shows encouraging preclinical efficacy in NSCLC. The second-generation proteasome inhibitor carfilzomib (CFZ) is superior to BTZ and overcomes BTZ resistance in multiple myeloma patients. Here, we show that CFZ together with NFV is superior to the BTZ + NFV combination in inducing endoplasmic reticulum stress and proteotoxicity through the accumulation of excess proteasomal substrate protein in NSCLC in vitro and ex vivo. Interestingly, NFV increases the intracellular availability of CFZ through inhibition of CFZ export from NSCLC cells that express multidrug resistance (MDR) protein. Combining CFZ with NFV may therefore represent a future treatment option for NSCLC, which warrants further investigation.

## Introduction

Lung cancer is among the deadliest cancers^1^. Worldwide every year, 1.8 million people are diagnosed with lung cancer, and 1.6 million die of the disease^2^. Its death rate exceeds that of colon, breast, and prostate cancers combined. Non-small-cell-lung carcinoma (NSCLC) accounts for 85% of all lung cancers^3^. Although personalized medicine targeting appropriate molecular targets has helped to improve survival in patients with NSCLC^4,5^, more than half of patients still die within one year of diagnosis, and the 5-year survival is approximately 17%^5,6^. Several reasons might be responsible for the poor prognosis. First, approximately 40% of newly diagnosed lung cancer patients are stage IV^5^. Second, the vast majority of NSCLC patients treated with chemotherapy for advanced disease relapse and develop resistance to conventional chemotherapy that targets the DNA replication machinery. Only a small proportion of NSCLC patients present with a known oncogenic driver mutation that can be pharmacologically targeted, and in these patients, molecular and clinical resistance almost invariably develops^6,7^. Third, although immunotherapy is very promising, only a fraction of NSCLC patients respond to checkpoint inhibitor monotherapy, and therapy resistance most often occurs^8^. Thus, despite these innovations, new approaches for the treatment of patients with NSCLC are highly warranted.

The ubiquitin proteasome system plays a central role in cellular protein homeostasis that is significantly altered in cancer cells, including lung carcinoma^9^. Inhibition of this system provokes the accumulation of misfolded proteins, resulting in proteotoxic stress. If this stress is not relieved, it may culminate in cell cycle arrest and apoptosis^10,11^. Therefore, causing proteotoxicity by targeting the ubiquitin proteasome system represents a tumor biology-driven anticancer strategy that may overcome resistance to conventional chemotherapy^9^.

Bortezomib (BTZ) was approved for the treatment of multiple myeloma in 2003 as the first reversible proteasome inhibitor^12,13^. Preclinical data showed encouraging cytotoxic efficacy of BTZ alone as well as in combination with other anticancer agents against lung cancer. However, the combination of BTZ with conventional chemotherapy showed low clinical efficacy^9^. Our previous preclinical and clinical data showed that the proteotoxic activity of BTZ can be significantly improved by combining it with nelfinavir (NFV), an HIV protease inhibitor known to induce proteotoxic stress in vitro and in vivo^14–16^. NFV alone induces stress in the endoplasmic reticulum (ER) and apoptosis in NSCLC^17^. The combination of NFV with BTZ showed encouraging synergistic preclinical activity in NSCLC^18^. BTZ and NFV induce ER stress and the unfolded protein response (UPR) through different mechanisms of action^16,19^, and their combination synergistically triggers the terminal UPR and induces apoptosis in multiple cancers^14,20,21^. The second-generation proteasome inhibitor carfilzomib (CFZ) is superior to BTZ and overcomes BTZ resistance in myeloma patients^22^. CFZ was clinically tested in combination with irinotecan in patients with relapsed small cell lung cancer, showing good tolerance and a promising response rate^23^. We hypothesized that CFZ may be even more potent than BTZ in inducing proteotoxicity in combination with NFV in NSCLC. Our results show that CFZ is more potent in inducing proteotoxicity and cytotoxic activity in combination with NFV than BTZ, likely at least in part through the inhibition of multidrug resistance pumps that mediate the efflux of CFZ from cancer cells.

## Results

### Cytotoxic activity of BTZ and CFZ as monotherapies in vitro

**Figure 1:**
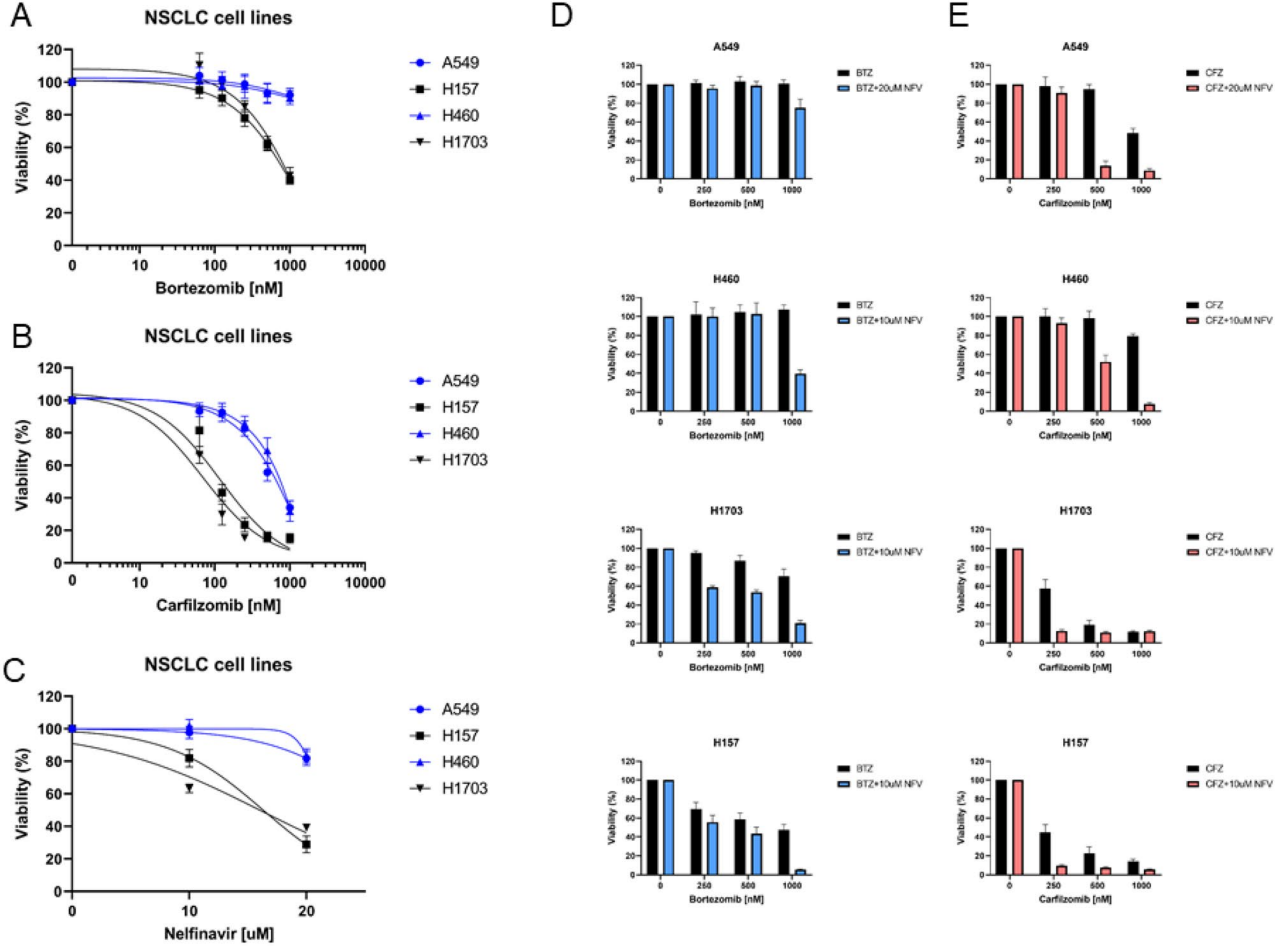
Cytotoxicity of proteasome inhibitors alone or in combination with nelfinavir in NSCLC cell lines. Cell viability assays (MTS) were performed with NSCLC cell lines A549 (adenocarcinoma), H460 (large cell lung cancer), H157 (squamous cell carcinoma) and H1703 (adenocarcinoma), and dose‒response curves were obtained to calculate the IC50 values for the drugs in all cell lines (presented in Table S1). Cells were treated with increasing concentrations of (**A**) bortezomib and (**B**) carfilzomib for two hours, washed, and then left in drug-free medium for an additional 48 h. (**C**) Cells were treated with increasing concentrations of nelfinavir continuously for 48 h. Data represent the mean ± SD of at least three replicates. Escalating doses of (**D**) bortezomib and (**E**) carfilzomib were added for 2 h. After washing away proteasome inhibitors, nelfinavir was added for an additional 48 h at a concentration of 10 µM, except for A549 cells, where a concentration of 20 µM was used. The synergistic effect for drug combinations was calculated, and the most significant synergy is presented in Table S2. Data represent the mean ± SD of at least three replicates.

To compare the cytotoxicity of BTZ and CFZ in NSCLC cell lines, cells were treated with escalating concentrations of BTZ and CFZ for two hours, washed, and then left in drug-free medium for 48 h (Fig. 1). In general, the adenocarcinoma cell line A549 and the large cell carcinoma line H460 were more resistant to both drugs than squamous cell lung carcinoma line H157 and adenocarcinoma line H1703 (Fig. 1A,B, Table S1). In A549 and H460 cells, only CFZ monotherapy showed significant cytotoxicity in a therapeutic drug range (CFZ: IC_50_ for A549 = 625 nM, for H460 = 706 nM), whereas the cells were insensitive to high doses of BTZ (BTZ: IC_50_ for A549 = 2108 nM, for H460 = 10,793 nM). H157 and H1703 were more sensitive to CFZ, as significant toxicity was induced at low drug doses (CFZ: IC_50_ for H157 = 126 nM, for H1703 = 85 nM), whereas BTZ at the highest dose induced approximately 50% cytotoxicity in both cell lines (BTZ: IC_50_ for H157 = 630 nM, for H1703 = 703 nM). Thus, CFZ monotherapy is more cytotoxic in all cell lines than BTZ. Notably, only CFZ induced higher cytotoxicity at concentrations that can be achieved in patient plasma.

### Cytotoxic activity of BTZ and CFZ combined with NFV in vitro

Next, the cytotoxicity of NFV alone and in combination with either BTZ or CFZ was assessed in the same set of NSCLC cell lines (A549, H460, H157 and H1703). As previously described, cells were treated with BTZ or CFZ for 2 h, then were washed out of proteasome inhibitors and left in drug-free medium or in NFV-containing medium for an additional of 48 h. To assess the NFV monotherapy-induced effect, the cells were treated continuously with NFV for 48 h. NFV alone was slightly more cytotoxic in the H157 and H1703 cell lines, and the same cells were more sensitive to BTZ and CFZ single-drug treatment (Fig. 1C). In the four cell lines tested, the combination of NFV and BTZ showed nearly no relevant additional cytotoxicity compared to BTZ alone (Fig. 1D). In contrast, the addition of NFV to CFZ significantly increased the cytotoxic effect of CFZ in all cell lines (Fig. 1E, Table S2). Remarkably, NFV induced additional cytotoxicity at concentrations of 10 µM (except for A549, where the necessary dose was 20 µM), a plasma concentration that is clinically achievable in patients^24^. The combination of CFZ and NFV showed stronger cytotoxicity than BTZ and NFV in all cell lines, which is reflected by the low combination indices of the CFZ combinations (Table S2).

### Cytotoxicity of BTZ and CFZ in combination with NFV ex vivo in patient-derived samples

**Figure 2:**
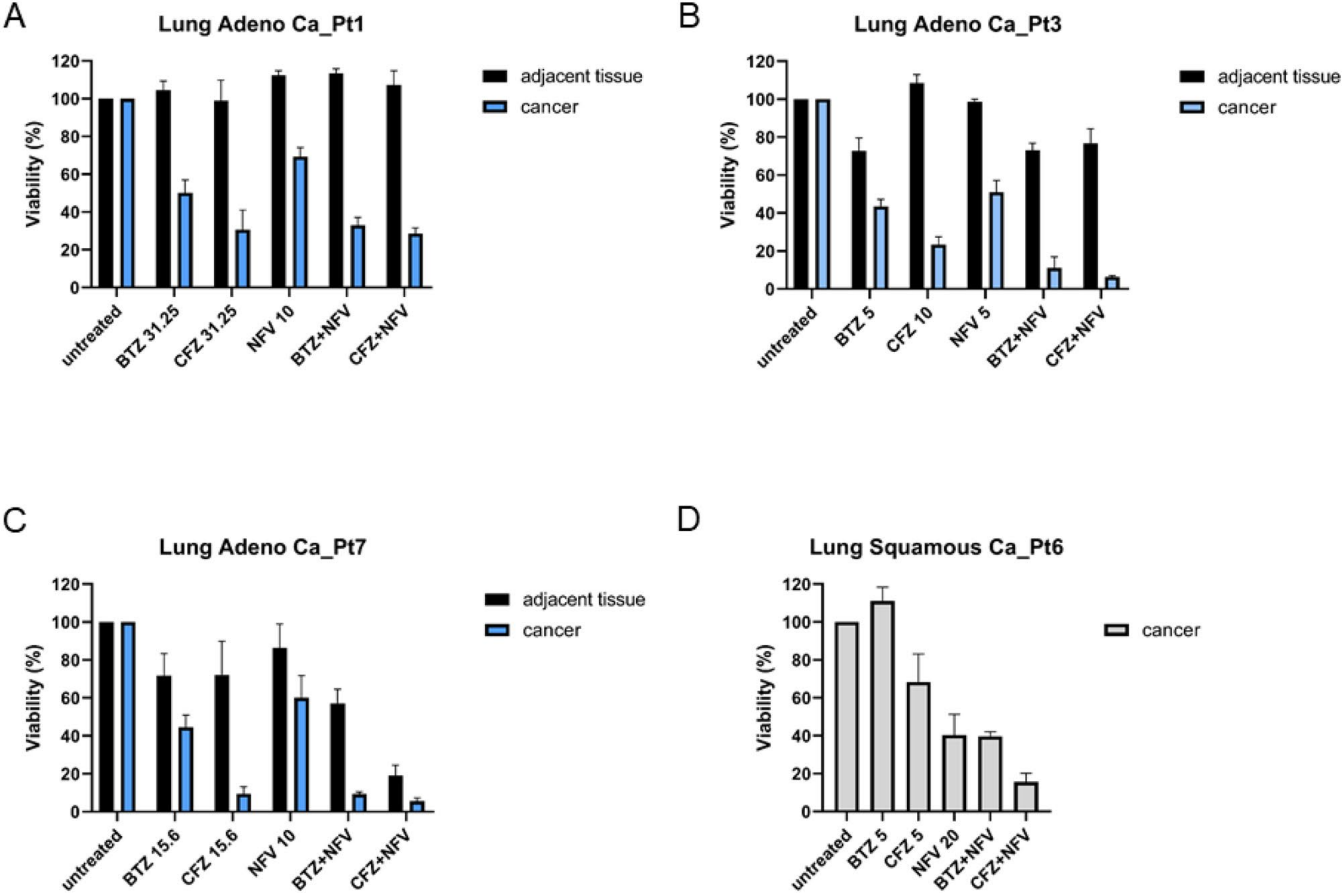
Cytotoxicity of proteasome inhibitors in combination with nelfinavir in NSCLC patient-derived primary cells. Primary cells derived from surgically resected tumors and adjacent healthy tissue from NSCLC patients were isolated, characterized by flow cytometry and subjected to cytotoxicity assays. The cells were exposed to bortezomib (nM), carfilzomib (nM) and nelfinavir (µM) in monotherapy or in combination continuously for 48 h. (**A**–**C**): adenocarcinoma and adjacent healthy lung tissue; (**D**) squamous cell carcinoma without adjacent healthy lung tissue.

To investigate whether CFZ or BTZ together with NFV is also effective on patient-derived primary cells, samples of tumor and adjacent healthy tissue were obtained from 4 patients undergoing resection of NSCLC (Table 1). Primary cells from adeno carcinomas and adjacent healthy tissue were isolated, characterized by flow cytometry and subjected to cytotoxicity assays with BTZ, CFZ, NFV and their combinations continuously for 48 h. The cytotoxic effect of CFZ alone was more pronounced than that of BTZ alone in primary adenocarcinoma cells, confirming the results obtained in cell lines (Fig. 2A–C). However, the combination of both CFZ and BTZ with NFV showed a synergistic effect, unlike in the cell lines, where BTZ was not synergistic with NFV, which is possibly due to prolonged treatment with the proteasome inhibitors for 48 h Patient-derived adjacent healthy lung cells were significantly less sensitive to this treatment. In the only patient-derived primary sample of squamous carcinoma, the cytotoxic effect of CFZ combined with NFV was more pronounced than that of the combination of BTZ and NFV (Fig. 2D). Unfortunately, in this sample, the effect on adjacent tissue could not be quantified.

**Table 1:** Baseline characteristics of NSCLC patients.

Patient	Histology	Gender	Age at operation (years)	Tumour stage	Previous treatment	Smoking history	Molecular alterations
1	Adenocarcinoma	Female	41	pT2, pN2 (02/16), G2, R0 pM1 (brain)	None	Yes, 15py	K-ras mutation p. G12C
							EGRF not mutated
							PDL1 expression negative
							MET not mutated
							ROS1 translocation negative
3	Adenocarcinoma	Male	46	pT1a, pN0 (0/18), G2, R0	None	Yes, 30py	Not tested
7	Adenocarcinoma	Female	49	pT2a, pN0 (0/19), G3, R0	None	Yes, 35py	Not tested
6	Squamous cell carcinoma	Male	67	pT4, pN1 (4/38), G2, R1	None	Yes, 50py	Not tested

py = pack year (cigarettes per day/pack size) × years).

### Induction of the UPR, ubiquitinated protein accumulation and apoptosis

**Figure 3:**
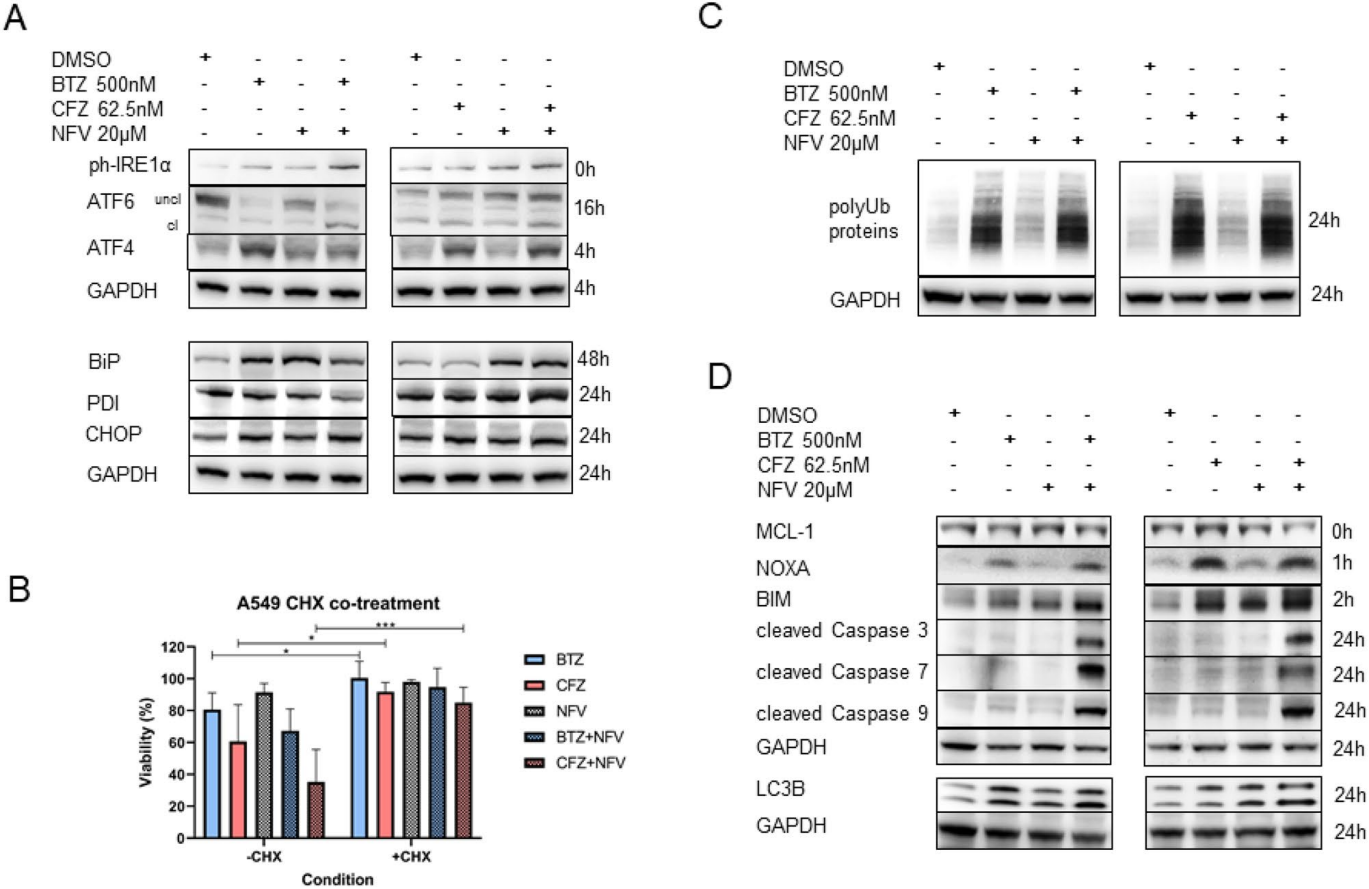
ER stress and apoptosis induction by proteasome inhibitors in combination with nelfinavir in NSCLC cells. (**A**) Induction of the proteins involved in the UPR upon treatment with bortezomib and carfilzomib alone or in combination with nelfinavir. Western blot analysis was performed with A549 cells at the indicated time points after 2 h of pulse treatment with proteasome inhibitors with or without nelfinavir. Representative images of three independent experiments are shown. (**B**) Cytotoxicity of bortezomib, carfilzomib and nelfinavir in the presence or absence of 1 µg/ml cycloheximide (CHX) pretreatment for 3 h. Cell viability was assessed 48 h after 2 h of pulse treatment with proteasome inhibitors, followed by incubation in drug-free media or nelfinavir. Data represent the mean ± SD of at least three replicates. (**C**) Accumulation of polyubiquitinated proteins upon treatment with bortezomib and carfilzomib alone or in combination with nelfinavir. Western blot analysis was performed with A549 cells at the indicated time points after 2 h of pulse treatment with proteasome inhibitors alone or in combination with nelfinavir. Representative images of three independent experiments are shown. (**D**) Induction of apoptosis upon treatment with bortezomib and carfilzomib alone or in combination with nelfinavir. Western blot analysis was performed with A549 cells at the indicated time points after 2 h of pulse treatment with proteasome inhibitors alone or in combination with nelfinavir. Representative images of three independent experiments are shown. * represents *p* < 0.05; *** represents *p* < 0.001.

Since both proteasome inhibitors and NFV induce ER stress that subsequently triggers UPR and their combination leads to terminal UPR and apoptosis induction, the induction of three branches of the UPR were analyzed in the A549 cell line. First, ATF6 that is cleaved in the Golgi apparatus into the active transcription factor; second, PERK-ph-eIF2α signaling, which is detectable by induction of the downstream transcription factor ATF4; and third, IRE1α/XBP1 axis induction was investigated. The phosphorylation of IRE1α leads to splicing of XBP1, which is a major switch to the UPR. The combination of BTZ and CFZ with NFV led to phosphorylation of IRE1α and ATF6 cleavage. Concerning ATF4, a higher induction of the PERK axis by CFZ and NFV was noted (Fig. 3A top). We further explored the status of BiP, the master regulator of ER stress, PDI as a folding chaperone, and CHOP as a link to ER stress-induced apoptosis (Fig. 3A bottom). The BiP protein level was upregulated by NFV and BTZ alone as well as by the combination of NFV and BTZ. CFZ alone induced BiP to a lesser extent than BTZ; however, the induction of BiP by CFZ and NFV was stronger than that by BTZ combined with NFV. Likewise, CFZ + NFV induced PDI, whereas the induction of PDI was not observed for BTZ + NFV treatment. In contrast, CHOP was also increased by BTZ alone and BTZ together with NFV, but the induction was more pronounced by the combination of CFZ with NFV.

Pretreatment of A549 cells for 3 h with 1 µg/ml cycloheximide, an inhibitor of protein synthesis, significantly abrogated the cytotoxic effect of BTZ and CFZ, as expected, but also the synergistic cytotoxic effect of CFZ and NFV. Likewise, cycloheximide abrogated the cytotoxicity of BTZ + NFV, but only mildly, as this combination also did not show a strong synergistic effect (Fig. 3B). Taken together, these data suggest that the combination of CFZ and NFV induces more pronounced ER stress due to impairment of protein homeostasis and turnover, leading to apoptosis.

To further test this hypothesis, we assessed the amount of polyubiquitinated proteins that line up for proteasomal degradation upon BTZ or CFZ treatment and in combination with NFV. NFV, as expected, did not induce the accumulation of polyubiquitinated proteins, consistent with our previous data showing that it induces ER stress due to impairment of membrane fluidity^16^. In contrast, CFZ induced more pronounced poly-Ub accumulation than BTZ, consistent with its different profile of proteasome β-subunits inhibition and stronger functional proteasome inhibition at higher doses that we observed in multiple myeloma^25^. The combination of BTZ or CFZ with NFV did not additionally increase the level of poly-Ub accumulation, which is in line with our previous observation in myeloma (Fig. 3C)^14^.

Subsequently, both BTZ and CFZ in combination with NFV induced apoptosis, which was reflected by the cleavage of caspases 3, 7 and 9 (Fig. 3D). Caspase 8 was of no relevance (data not shown). The proapoptotic BCL-2 family proteins Bim and Noxa were likewise increased upon BTZ + NFV and CFZ + NFV treatment; however, a stronger induction was observed for the CFZ + NFV combination and likewise for CFZ over BTZ monotherapy. Likewise, the CFZ + NFV combination led to a reduction in the level of MCL-1, an anti-apoptotic protein, which was not observed for the BTZ + NFV combination. Interestingly, the data suggest that autophagy (LC3B as a marker), as a rescue mechanism to proteasome inhibition, is induced by both BTZ and CFZ; however, the induction was stronger by BTZ monotherapy than CFZ. Subsequently, both BTZ and CFZ in combination with NFV induced LC3B protein levels. To determine if caspase activation is necessary for cell death in the combination setting, cells were pretreated with 50 µM Z-VAD (general caspase inhibitor) for 1 h. Z-VAD only mildly and not significantly abrogated the synergistic cytotoxicity of the CFZ + NFV combination (Fig. S1), suggesting that cell death induced by NFV with either BTZ or CFZ involves caspase-dependent as well as caspase-independent processes. Taken together, the data suggest that the mechanism of UPR induction provided by NFV differs from the one induced by CFZ and is independent on the inhibition of proteasomal degradation, but synergizes with CFZ in the cell toxicity.

### Proteasome inhibition

**Figure 4:**
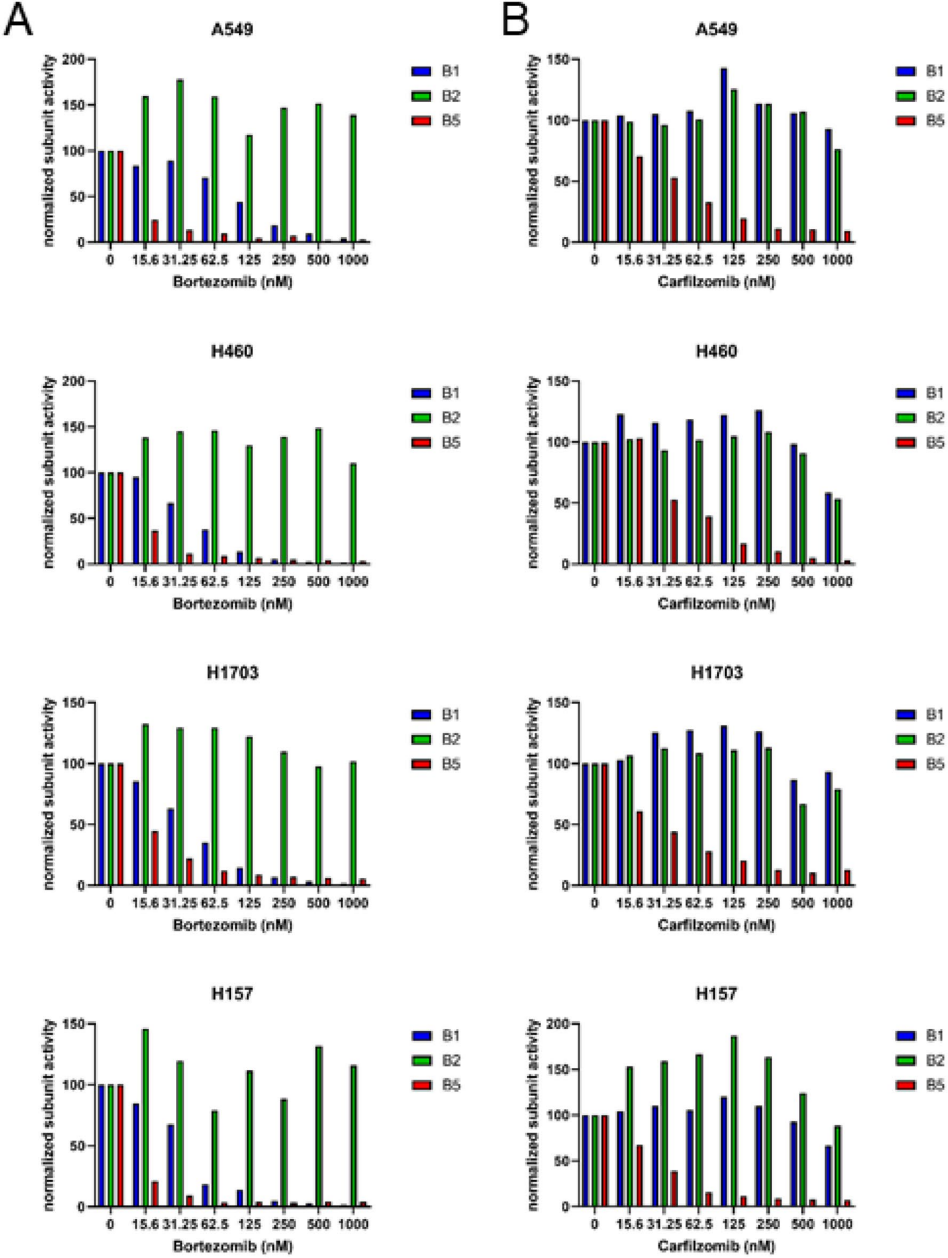
Inhibition of proteasome activity by bortezomib and carfilzomib in NSCLC cell lines. Active site labeling with proteasome subunit-selective probes was performed after (**A**) bortezomib or (**B**) carfilzomib pulse for 1 h in A549, H157, H460 and H1703 cell lines. Proteasome subunits are illustrated in different colors as follows: β1 in blue, β2 in green and β5 in red. Data represent the quantification of one representative experiment out of 3 replicates. The original data are presented in Fig. S2.

To further investigate the mechanism for the stronger accumulation of polyubiquitinated proteins by CFZ in NSCLC, we explored the proteasome activity after mono-treatment with either BTZ or CFZ in the four cell lines. Cells were treated with increasing concentrations of BTZ or CFZ for 1 h and subsequently labeled with fluorescently tagged proteasome subunit-selective probes, which covalently bind to the active center of the constitutive proteasome subunits β1, β2 and β5 and likewise to the immunoproteasome subunits β1i, β2i and β5i, which are present mostly in the immune cells. As expected, BTZ preferentially targets β5 (red) proteasome activity at lower doses and β1 (blue) activity at slightly higher doses (Fig. 4A and Fig. S2). At a concentration of 31.25 nM BTZ, the β5 subunits were completely inhibited in all four tested cell lines, and the β1 subunits were completely inhibited at a 125 nM BTZ dose. In contrast, CFZ targets only the β5 subunit at lower doses, but unlike BTZ, it co-inhibits the β2 subunit at higher doses (Fig. 4B and Fig. S2), which is consistent with our observations in multiple myeloma^25^. CFZ fully inhibited β5 subunits at a higher concentration (125 nM) than BTZ and started to inhibit the β2 subunit at a dose of approximately 1000 nM. Overall, the profile of proteasome inhibition by BTZ and CFZ reflects the published data in multiple myeloma^25^. However, it does not explain the observed cytotoxicity of CFZ at a lower dose.

**Figure 5:**
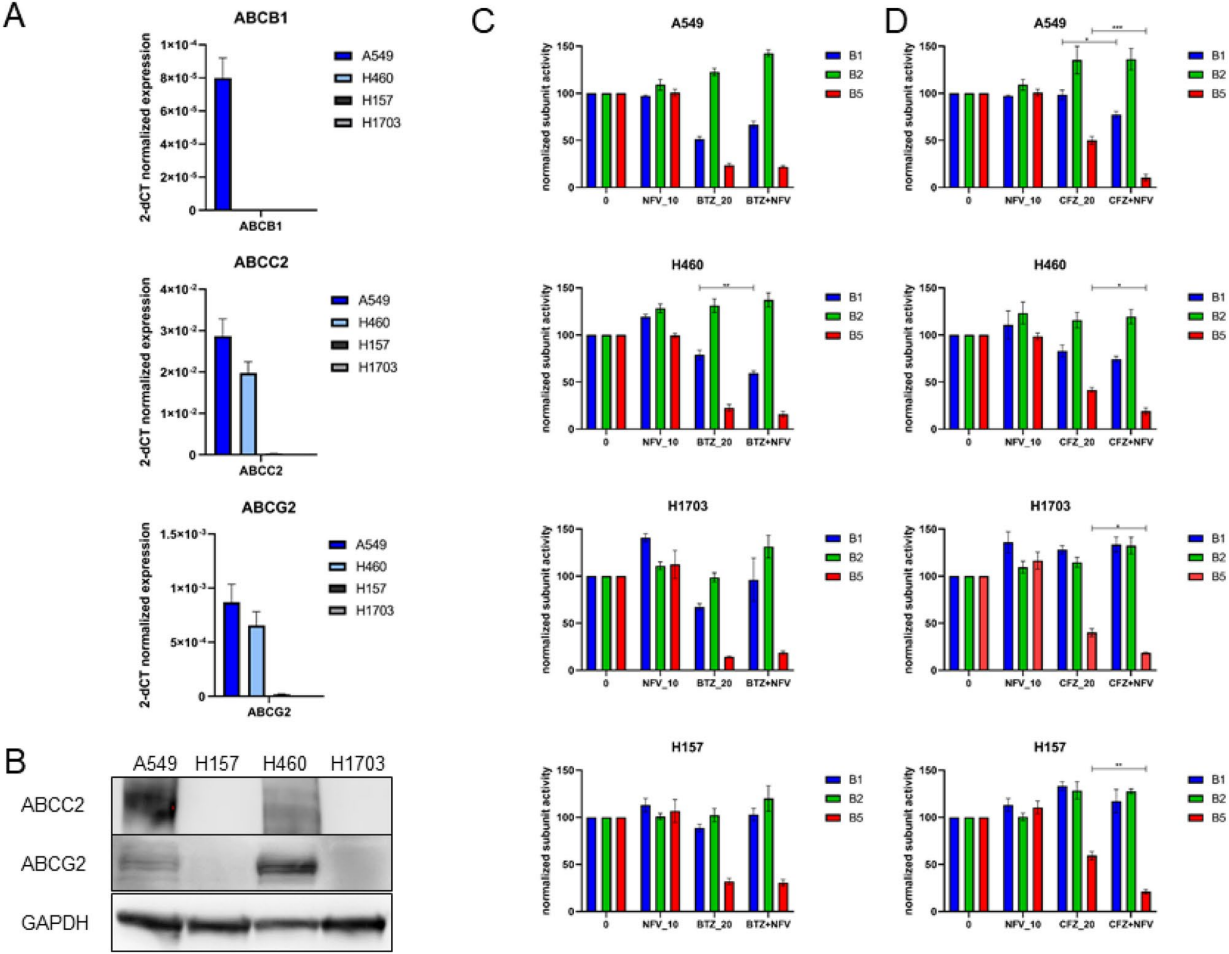
Efflux pumps and their involvement in the intracellular availability of carfilzomib in NSCLC cell lines. (**A**) Expression of ATP-type transporters ABCB1, ABCC2 and ABCG2 in A549, H157, H460 and H1703 cell lines as determined by quantitative real-time PCR. Data are normalized to GAPDH, expressed in 2^−dCt^ values and are presented as the mean ± SD of at least 3 replicates. (**B**) Levels of ATP-type transporters ABCC2 and ABCG2 in the same cell lines determined by western blot. Data show a representative blot of 3 replicates. (**C**, **D**) Active site labeling with proteasome subunit-selective probes was performed after (**C**) bortezomib or (**D**) carfilzomib pulse treatment (both drugs 10 nM and 20 nM) alone or in combination with nelfinavir (20 µM for A549; 10 µM for H460, H157 and H1703) for 1 h in NSCLC cell lines. Proteasome subunit activities are illustrated in different colors as follows: β1 in blue, β2 in green and β5 in red. Data represent the mean ± SD of at least two replicates. The original data are presented in Fig. S3. * Represents *p* < 0.05; ** represents *p* < 0.01; *** represents *p* < 0.001.

To further investigate the underlying mechanism of synergistic cytotoxicity between CFZ and NFV, the expression of multidrug resistance transporters, of which CFZ is a strong substrate, were analyzed. ABCB1 (ATP binding cassette, subfamily B, member 1), also known as multidrug transporter (MDR1) or P-glycoprotein (P-gp), which has been shown to be present in CFZ-resistant cells^26,27^, is rather poorly expressed in all four NSCLC lines used in the present study (Fig. 5A). However, importantly, it is detectable in the A549 cell line in which CFZ and NFV showed the strongest synergistic cytotoxicity. In contrast, two other membrane transporters, ABCC2 and ABCG2 (BCRP), were found to be expressed in A549 and H460, the cell lines that are generally more resistant to BTZ and CFZ. These membrane transporters were absent in the proteasome inhibitor-sensitive cell lines H157 and H1703 (Fig. 5A,B). Nevertheless, the presence of other transporter complexes, for which CFZ may be a substrate, cannot be excluded. Previously, we showed that NFV inhibits ABCB1 efflux activity^26^. Thus, we hypothesized that in addition to ER stress induction, NFV increases the intracellular concentration of CFZ to provide stronger proteasome inhibition. We used active site proteasome labeling to show the functional effect of NFV on extracellular transporters with regard to residual proteasome activity in all four cell lines (Fig. 5C,D and Fig. S3). NFV co-treatment did not change the profile of proteasome inhibition for BTZ in the four tested cell lines (Fig. 5C). In contrast, NFV increased the intracellular availability of CFZ for proteasome inhibition, as reflected by significantly lower residual β5 activity (red bands) in CFZ + NFV co-treatment than CFZ monotherapy (Fig. 5D). The most significant effect was again observed in the A549 cell line, which expresses ABCB1. These data indicate that NFV increases the intracellular availability of CFZ for proteasome inhibition in NSCLC cells, and subsequent more effective proteasome inhibition is one of the mechanisms underlying the synergistic cytotoxic effect between NFV and CFZ.

## Discussion

Here, we show that both BTZ and CFZ in combination with NFV have a cytotoxic effect on NSCLC cell lines, while the combination of CFZ with NFV showed a more pronounced in vitro cytotoxicity than BTZ + NFV. In primary NSCLC cells, CFZ monotherapy induced a higher cytotoxicity than BTZ alone. Although both combinations were equally effective in killing adenocarcinoma cells from patient samples, the cytotoxic effect of CFZ together with NFV was stronger in squamous carcinoma cells. Adjacent healthy tissue of the lung was less affected than tumor cells by either therapy.

In accordance with the present findings, Kawabata et al. demonstrated earlier that BTZ together with NFV synergistically induced cell death in NSCLC cells due to dual induction of ER stress^18^. Although the mechanism of ER stress induction and accumulation of polyubiquitinated proteins in the cytosol by both drugs differ, their effect culminated in cytosolic proteotoxicity^18^. According to the present findings, the in vitro and to some extent ex vivo cytotoxic activity of CFZ together with NFV was superior to that of the BTZ + NFV combination. Although both proteasome inhibitors in combination with NFV induced ER stress, only CFZ + NFV induced ATF4 and BiP protein expression, reflecting stress-response pathway induction. Moreover, NFV potentiated the proteasome-inhibitory effect only in combination with CFZ, whereas for BTZ, no such effect was observed. Surprisingly, when looking at proteasome inhibition by both drugs in monotherapy, BTZ inhibited its target, the β5 proteasome subunit, at a much lower drug concentration than CFZ. In contrast, NFV increased proteasome inhibition caused by CFZ to a level comparable or even stronger to the equimolar dose of BTZ. This apparent discrepancy is explained by the fact that CFZ, in contrast to BTZ, is a strong substrate of ABCB1 (p-glycoprotein) and other drug efflux transporters^26^. ABCB1-mediated export of CFZ limits its cytotoxic effect. NFV is able to reduce the activity of these efflux pumps by thus far not fully understood mechanisms involving membrane fluidity^16^. ABCB1, the most studied multidrug transporter, was expressed at rather low levels in the four NSCLC cell lines used in the present investigation. In contrast, two other membrane pumps, ABCC2 and ABCG2 (BCRP), were found to be expressed in A549 and H460 cells, which are more resistant to BTZ and CFZ. These two membrane pumps were absent in the proteasome inhibitor-sensitive cell lines H157 and H1703, suggesting that they are involved in a general resistance to proteasome inhibitors and not specifically to CFZ. Nevertheless, NFV likely blocks the efflux of CFZ via another, not yet determined transporter, leading to increased intracellular CFZ concentration and stronger β5 proteasome subunit inhibition. Therefore, CFZ together with NFV not only induce relevantly more ER stress but also provide a stronger proteasome inhibition, both culminating in overwhelming proteotoxic stress leading to cell death. These data are supported by the fact that pretreatment with the protein synthesis inhibitor cycloheximide significantly decreased cell death caused by CFZ + NFV but to a lesser extent by BTZ + NFV, indicating that inhibition of protein synthesis increases cell survival by reducing proteotoxic stress. While the synergistic induction of proteotoxicity by CFZ + NFV was clearly superior to BTZ + NFV in the cell lines tested, this was less evident in patient-derived primary NSCLC cells. This may be explained by the fact that patient biopsies were retrieved from treatment-naïve patients with NSCLC, while the expression of MDR proteins that contribute to the synergistic activity of CFZ and NFV in NSCLC are relatively poorly expressed in treatment-naïve NSCLC and are significantly more highly expressed in chemotherapy-resistant NSCLC after several lines of systemic therapy^28,29^.

Conventional chemotherapy and novel targeted treatments cannot cure NSCLC due to intrinsic or emergent drug resistance^6–8^. Thus, new therapy options are still an urgent medical need. Disturbance of protein homeostasis with proteasome inhibitors represents an alternative treatment approach, which has been clinically proven to be effective in multiple myeloma^30,31^. However, although BTZ has been extensively tested in various cancers, the clinical results and its anticancer efficacy for solid tumors have been rather disappointing. This is perhaps not surprising, considering that myeloma cells are more sensitive to disturbance of protein homeostasis due to the massive production of monoclonal immunoglobulins and strong dependence on a degradation machinery to balance the induced ER stress^32^. The majority of other cancer types also show a higher protein turnover because of their increased growth rate and genetic instabilities but to a much lower level compared to multiple myeloma. Thus, these cancer cells have a larger ER stress buffering capacity, and triggering the UPR has mostly a pro-survival effect. To induce apoptosis in these “low-level ER stress-type” cancers, such as lung cancer, it may be essential to combine proteasome inhibitors with an additional inductor of the UPR. Since the HIV protease inhibitor NFV is a clinically approved drug able to induce the UPR^14^, its combination with proteasome inhibitors has the potential to induce overwhelming ER stress, resulting in a switch from pro-survival to pro-apoptotic UPR and subsequent apoptosis of tumor cells. Subsequently, the identification of an ideal and most effective proteasome inhibitor as a combination partner to NFV is important prior to the clinical testing of both drugs in NSCLC. In this regard, CFZ as a second-generation proteasome inhibitor shows efficacy in BTZ-resistant samples and is highly selective for the proteasome, showing minimal off-target toxicity^22^. Unlike BTZ, treatment with CFZ shows much lower rates of cumulative neurotoxicity, and thus, its treatment is not associated with dose-limiting side effects^33^. Therefore, treatment with CFZ over a prolonged period, e.g., until progression, is feasible. At the same time, compared to high-dose BTZ, CFZ at high doses provides a different inhibition profile of the proteasome, leading to more effective functional proteasome inhibition^25^. Besse et al. comprehensively demonstrated in a head-to-head comparison of the different and currently available proteasome inhibitors that β5 and β2 subunit coinhibition is the most effective proteasome inhibition profile, showing higher cytotoxicity. Full β5/β2 subunit inhibition was only reached by CFZ at higher concentrations (< 30 mg/m^2^) but not by BTZ^25^. However, as a good substrate of MDR transporters and having low tissue penetration, CFZ is not ideal as a single agent to be used in solid tumors^25^. Considering these facts, together with the results of the present investigation, CFZ in combination with NFV is a promising combination, as it may not only be more clinically effective but also possibly be associated with less unfavorable side effects when applied in higher doses.

In conclusion, the combination of the second-generation proteasome inhibitor CFZ together with the HIV protease inhibitor NFV shows strong synergistic effects in NSCLC cell lines as well as in NSCLC patient-derived tumor cells ex vivo. Importantly, adjacent healthy tissue is less affected by the treatment combination, enabling a therapeutic window. CFZ together with NFV may represent a new, promising treatment option for systemic therapy of NSCLC, whose efficacy warrants further in vivo and clinical investigation.

## Material and methods

### Cell lines and inhibitors

NSCLC cell lines of different origins were used to mirror different types of NSCLC: A549: adenocarcinoma from alveolar type II pneumocytes of the human lung, H157: squamous cell lung carcinoma, NCI-H460: large cell lung cancer, NCI-H1703: adenocarcinoma. They were all purchased from ATCC and maintained in 10% FCS-supplemented RPMI-1640 with 1% penicillin/streptomycin (all Sigma‒Aldrich, Buchs, Switzerland). All cell lines were routinely tested for mycoplasma contamination using the MycoAlert Mycoplasma Detection Kit (Lonza, Basel, Switzerland) and were authenticated by STR-typing (DSMZ, Braunschweig, Germany). BTZ was provided by Ortho Biotech, Neuss, Germany, and CFZ was provided by Onyx Pharmaceuticals, Inc. (South San Francisco, CA, USA). NFV was kindly provided by the NIH AIDS Reagent Program.

### Viability assays

To determine cytotoxicity, CellTiter 96® AQueous One Solution cell proliferation assay (MTS, Promega) was used for cell lines, whereas CellTiter-Glo® 2.0 (CTG, Promega) was used for primary cells, due to its increased sensitivity, according to the manufacturer’s technical manual. Cells were seeded in 96-well plates and allowed to attach overnight. Thereafter, BTZ or CFZ was added in increasing concentrations for 2 h to mimic the inhibitors’ actions in patients. Following the *i.v.* bolus injection, there was hardly any inhibitor left in the plasma after 2 h^34^. After washing away BTZ and CFZ, NFV was added for an additional 48 h at a concentration of 10 µM, except for A549 cells, where a concentration of 20 µM was used.

### Determination of proteasome activity by active site labeling

Proteasome-specific affinity-based probes, which each target a specific proteasome subunit, were used to evaluate the activity of all constitutive and immunoproteasome subunits as described previously^35^. In brief, equal amounts of total cellular protein were resolved by SDS‒PAGE using bis–tris 12% gels (Thermo Fisher Scientific, MA, USA), and labeled active β-subunits were directly visualized in the gel by Fusion Solo S. Proteasome β-subunit-specific fluorescence signals were quantified using Bio 1D software (Vilber Lourmat). Labeling experiments were repeated at least twice in an independent fashion, and representative results are displayed.

### Western blots

Following SDS‒PAGE using Bis–Tris 10% gels (Thermo Fisher Scientific, MA, USA), proteins were directly blotted onto PVDF membranes. Subsequently, the membranes were blocked with RotiBlock (Roth, Germany) and incubated with the following antibodies: anti-ABCC2 (12559; CST, Danvers, MA, USA), anti-ABCG2 (42078; CST, Danvers, MA, USA), anti-ATF4 (10835; Proteintech), anti-ATF6 (15794; Proteintech), anti-BIM (2933; CST, Danvers, MA, USA), anti-BiP (Grp78; 610978; BD Biosciences), anti-cleaved caspase-3, -7, -9, (9644; 8438; 7237; all CST, Danvers, MA, USA) anti-CHOP (Gadd 153; sc-793; Santa Cruz, USA), anti-phospho-IRE1α (phospho S724; ab124945; abcam), anti-LC3B (3868; CST, Danvers, MA, USA), anti-MCL-1 (4572; CST, Danvers, MA, USA) anti-PDI (610946; BD Biosciences), anti-NOXA (OP180; Calbiochem), anti-polyUb (PW 8805-0500; Enzo, NY, USA), and anti-GAPDH (hrp-60004; Proteintech).

### qPCR

Total RNA was isolated from NSCLC cell lines using TRIzol (Ambion/Thermo Fisher Scientific) and Direct-zol RNA MiniPrep (Zymo Research, Irvine, CA, USA), followed by reverse transcription with a High-Capacity cDNA Reverse Transcription Kit (Applied Biosystems/Thermo Fisher Scientific). cDNA was used in duplex real-time PCR on a Light Cycler II instrument (Roche, Switzerland) using Gene Expression Master Mix and TaqMan specific assays for ATP-binding cassette subfamily B member 1 (ABCB1), ATP-binding cassette subfamily C member 2 (ABCC2), ATP-binding cassette subfamily G member 2 (ABCG2) and GAPDH as an internal control (all Applied Biosystems/Thermo Fisher Scientific).

### Patient samples

Samples (tumor and adjacent healthy tissue) were obtained from patients with NSCLC undergoing surgical resection after written informed consent was obtained and the baseline characteristics are depicted in Table 1. The study was approved by the local ethical committee within the frame of the St. Gallen Lung Biopsy Biobank project (EKSG 11/044/1B). All procedures were in accordance with the Declaration of Helsinki and the research was performed in accordance with relevant guidelines/regulations. The tissue was processed into a single-cell suspension using a gentleMACS™ Dissociator (Miltenyi Biotec) according to the manufacturer’s protocol. Thereafter, the single-cell suspension was characterized by flow cytometry analysis using surface staining with antibodies against CD326/EpCAM-FITC (Miltenyi), CD 45-PeCy7, and CD31/PECAM-1-APC (both from DB Bioscience). Epithelial tumor cells were selected and thereby enriched using anti-EpCAM microbeads (Miltenyi, Biotec). Sorted EpCAM-positive cells were subsequently used for cell viability assays.

### Statistical analysis

Statistical evaluation was performed in GraphPad Prism v.8 (GraphPad Software, La Jolla, CA, USA). For comparison of two groups, an unpaired t test was used. For a group comparison, two-way ANOVA was used. *P* values < 0.05 were considered statistically significant. Synergism between BTZ, CFZ and NFV was calculated using combination indices (CI)^36^, where a CI < 1 indicates synergism, and a CI > 1 indicates antagonism. Normalized isobolograms were produced by plotting the BTZ ratio (monotherapy dose vs. dose needed in combination to reach the same effect) on the x-axis versus the NFV ratio on the y-axis.

## Supplementary Information

Supplementary Information 1.

Supplementary Information 2.

Supplementary Information 3.

## Data Availability

The datasets generated during the current study are available from the corresponding authors upon reasonable request.
