# Supplementary material for: The cytotoxic activity of carfilzomib together with nelfinavir is superior to the bortezomib/nelfinavir combination in non-small cell lung carcinoma
Source: Sci Rep. 2023 Mar 17;13:4411. doi: 10.1038/s41598-023-31400-6 (PMC10023769; doi:10.1038/s41598-023-31400-6)
Supplement: Supplementary file 1 — Supplementary Information 1. [file 41598_2023_31400_MOESM1_ESM.pdf]

The cytotoxic activity of carfilzomib together with nelfinavir is superior to the bortezomib/nelfinavir combination in non-small cell lung carcinoma

Lenka Besse, Marianne Kraus, Andrej Besse, Christoph Driessen, Ignazio Tarantino

Supplementary figures:

Figure S1: Effect of a caspase inhibitor on the cytotoxic effect of proteasome inhibitors alone or in combination with nelfinavir. Cytotoxicity of bortezomib, carfilzomib and nelfinavir in the presence or absence of 50 μM Z-VAD (general caspase inhibitor) pretreatment for 1 hour. Cell viability was assessed after 48 h after 2 hours of pulse treatment with proteasome inhibitors followed by incubation in drug-free media or in media containing 20 μM nelfinavir.

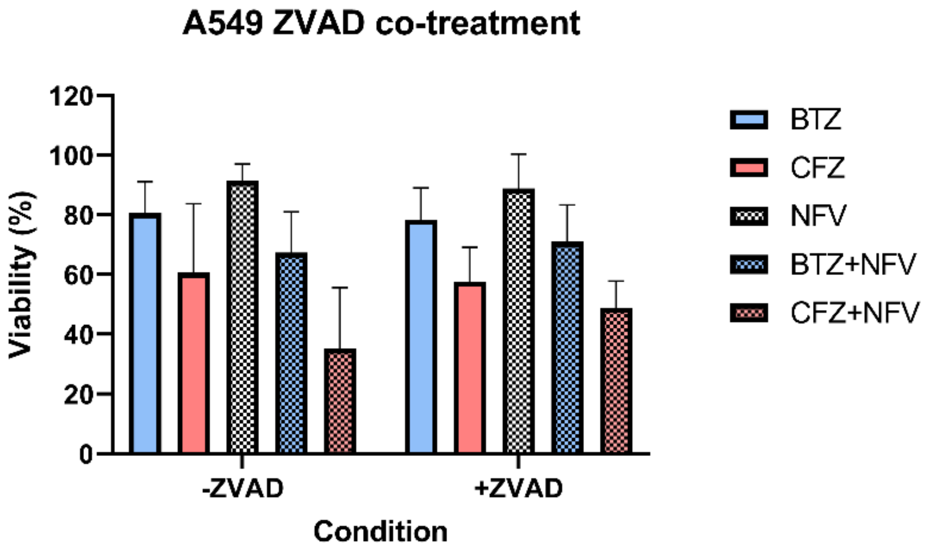

Figure S2: Active site labeling of proteasome activity after treatment with proteasome inhibitors. Activity-based proteasome probes were used to determine the residual activity of the proteasome after 1 hour of pulse treatment with bortezomib or carfilzomib in four NSCLC cell lines. Residual activity of the β2 subunit is presented in green (highest band), residual β1 activity is presented in blue (middle band), and residual β5 activity is presented in red (lowest band).

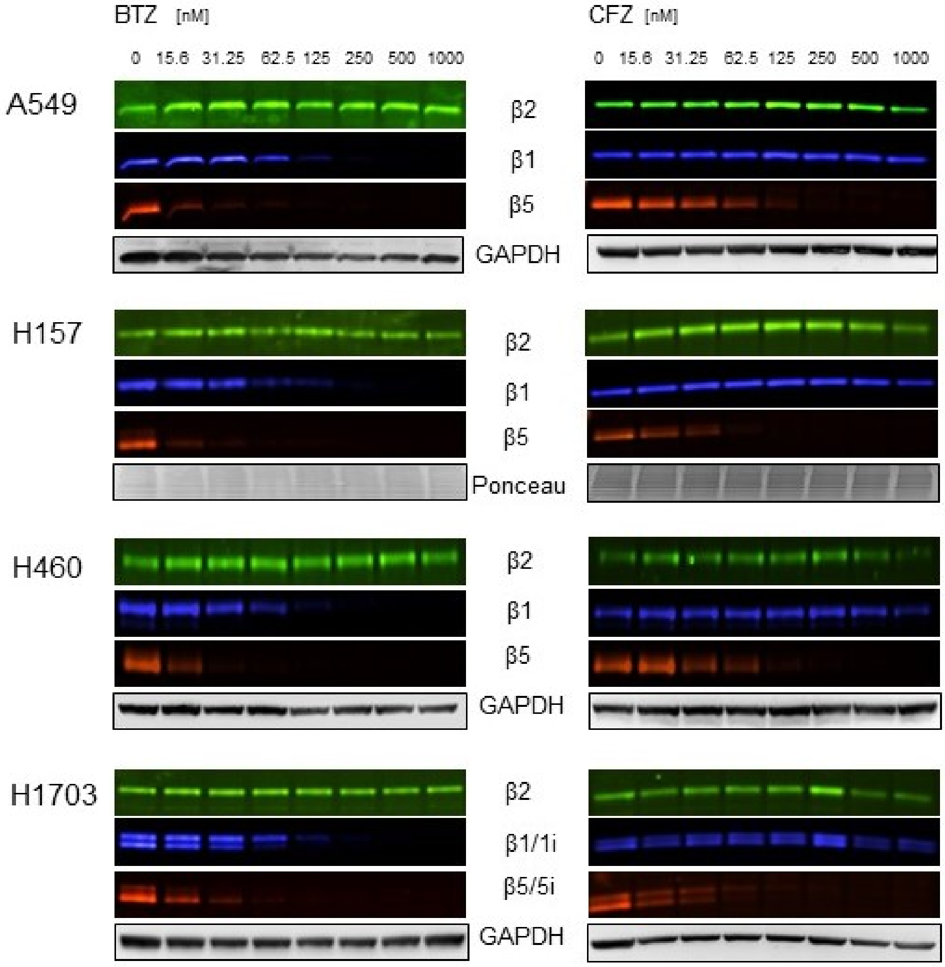

Figure S3: Active site labeling of proteasome activity after treatment with proteasome inhibitors and nelfinavir. Activity-based proteasome probes were used to determine the residual

activity of the proteasome after 1 hour of pulse treatment with bortezomib (B; in nM) or carfilzomib C (C; in nM) alone or in combination with nelfinavir (N; in μM) in four NSCLC cell lines. Residual activity of the β2 subunit is presented in green (highest band), residual β1 activity is presented in blue (middle band), and residual β5 activity is presented in red (lowest band).

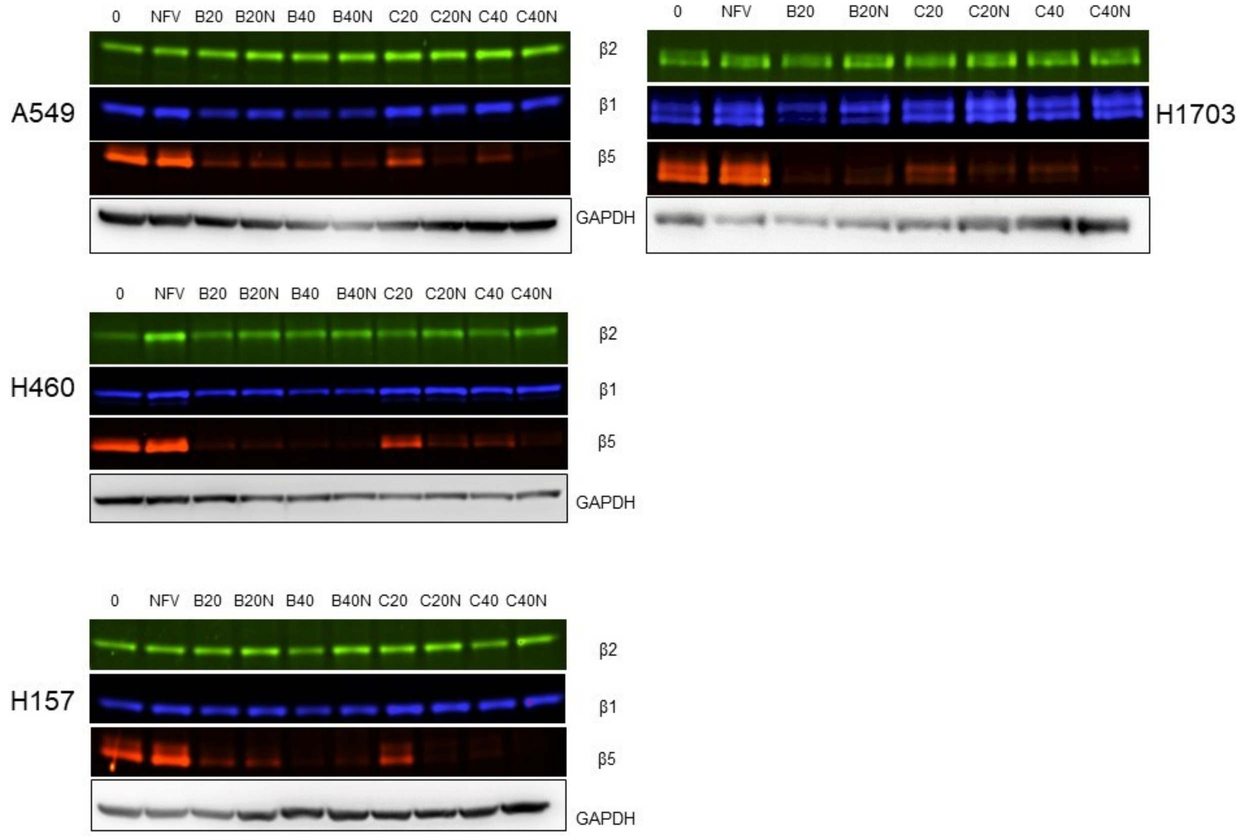

Supplementary tables:

Table S1: IC_{50} values obtained from the dose response curves presented in Figure 1A-C

IC50	A549	H157	H460	H1703
**Bortezomib (nM)**	2108	630.6	10793	702.5
**Carfilzomib (nM)**	625.2	126.3	705.5	85.51
**Nelfinavir (uM)**	26.56	16.31	21.37	16.02

Table S2: Highest combination indices from the combination treatments presented in Figure 1D

	CI Index		Drug dose
**Cell lines**	BTZ + NFV (10 μM)	CFZ + NFV (10 μM)	BTZ/CFZ (nM)
**A549**	0.312	**0.014**	500
**H460**	0.211	**0.059**	500
**H1703**	0.229	**0.087**	250
**H157**	0.7	**0.074**	250
