# Supplementary material for: The cytotoxic activity of carfilzomib together with nelfinavir is superior to the bortezomib/nelfinavir combination in non-small cell lung carcinoma
Source: Sci Rep. 2023 Mar 17;13:4411. doi: 10.1038/s41598-023-31400-6 (PMC10023769; doi:10.1038/s41598-023-31400-6)
Supplement: Supplementary file 2 — Supplementary Information 2. [file 41598_2023_31400_MOESM2_ESM.pdf]

Originals for Fig S2

A549

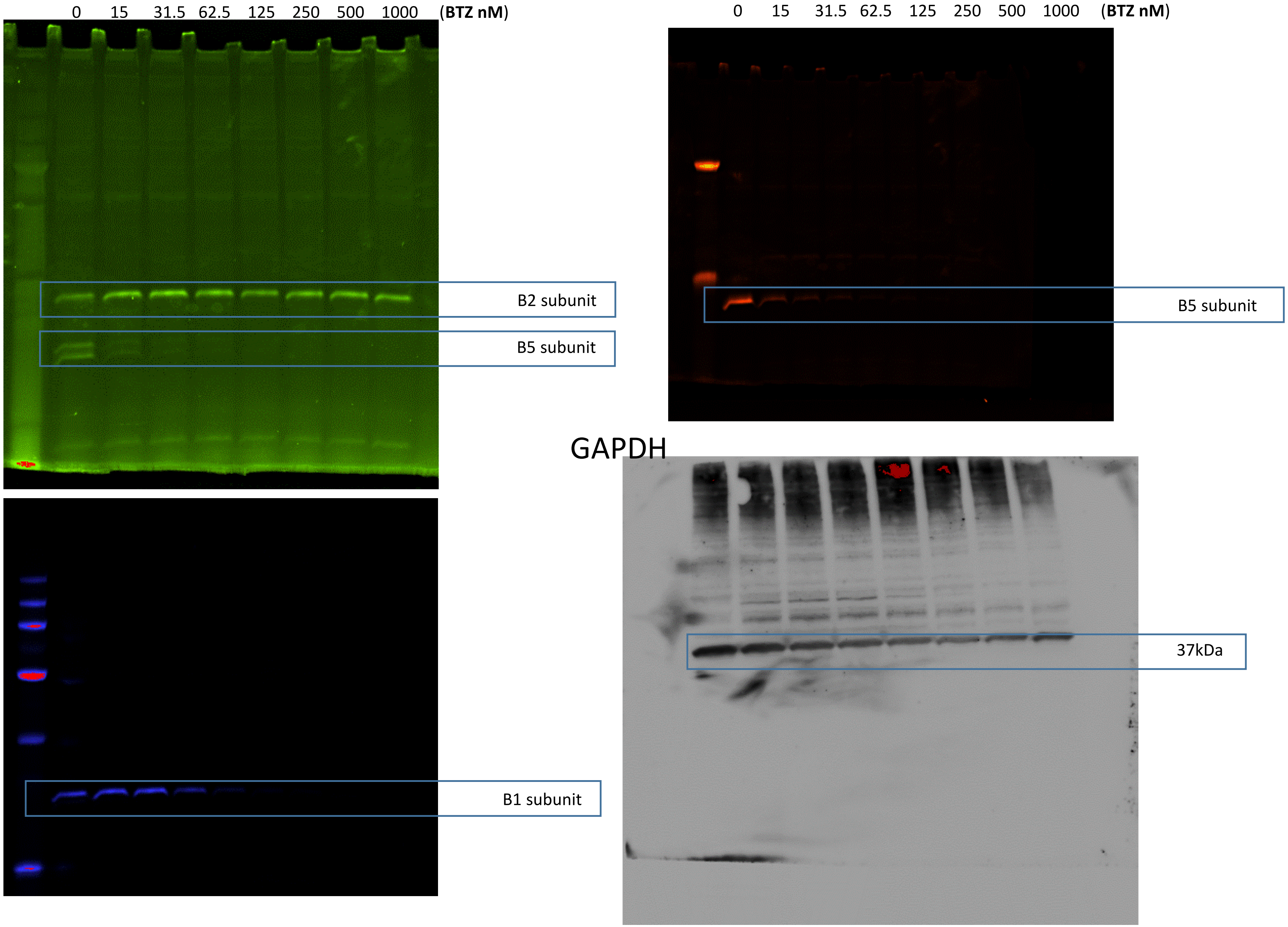
0 15 31.5 62.5 125 250 500 1000 (BTZ nM)
B2 subunit
B5 subunit
B1 subunit
0 15 31.5 62.5 125 250 500 1000 (BTZ nM)
B5 subunit
GAPDH
37kDa

A549

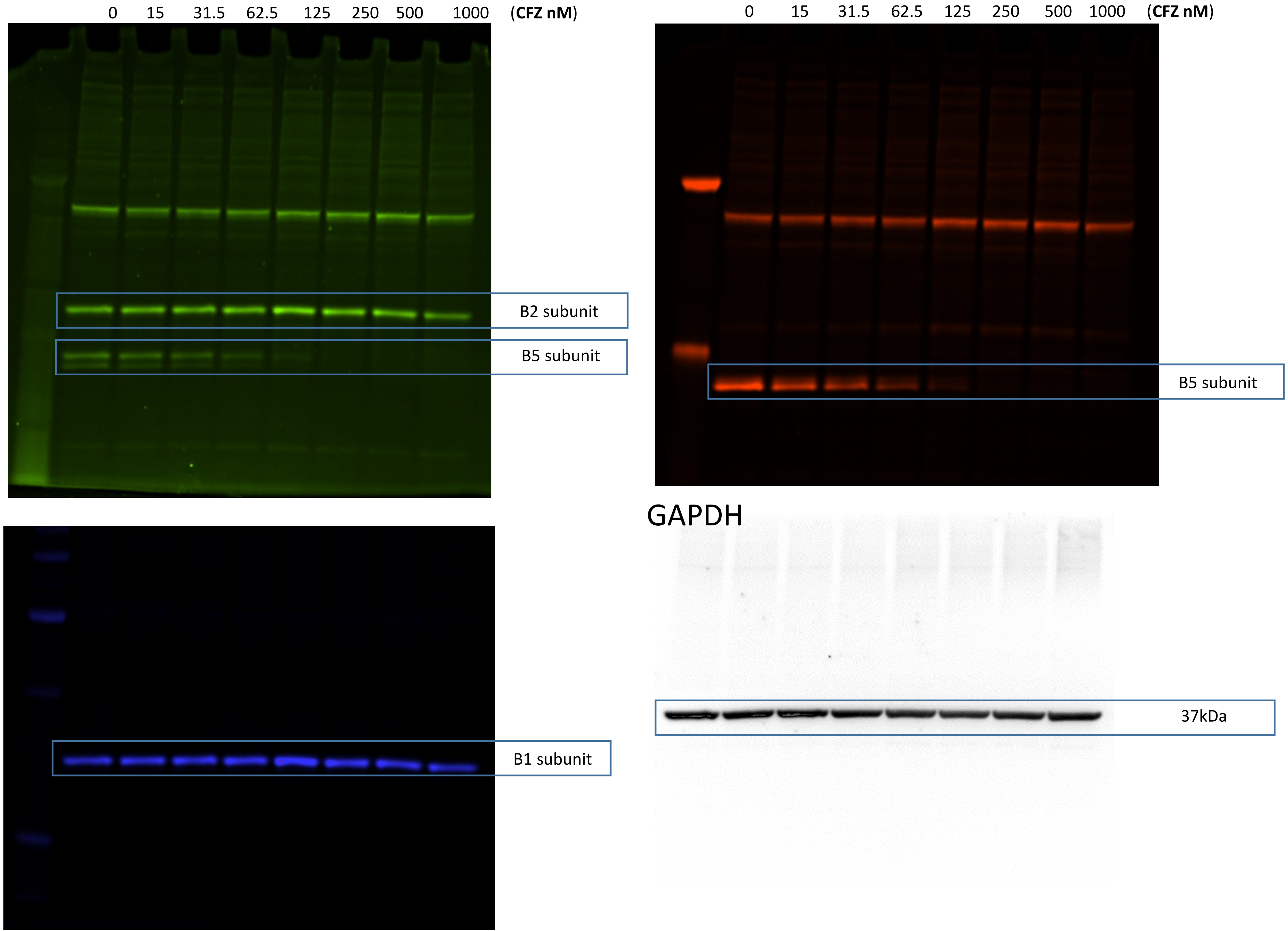
0 15 31.5 62.5 125 250 500 1000 (CFZ nM)
B2 subunit
B5 subunit
0 15 31.5 62.5 125 250 500 1000 (CFZ nM)
B5 subunit
B1 subunit
GAPDH
37kDa

H157

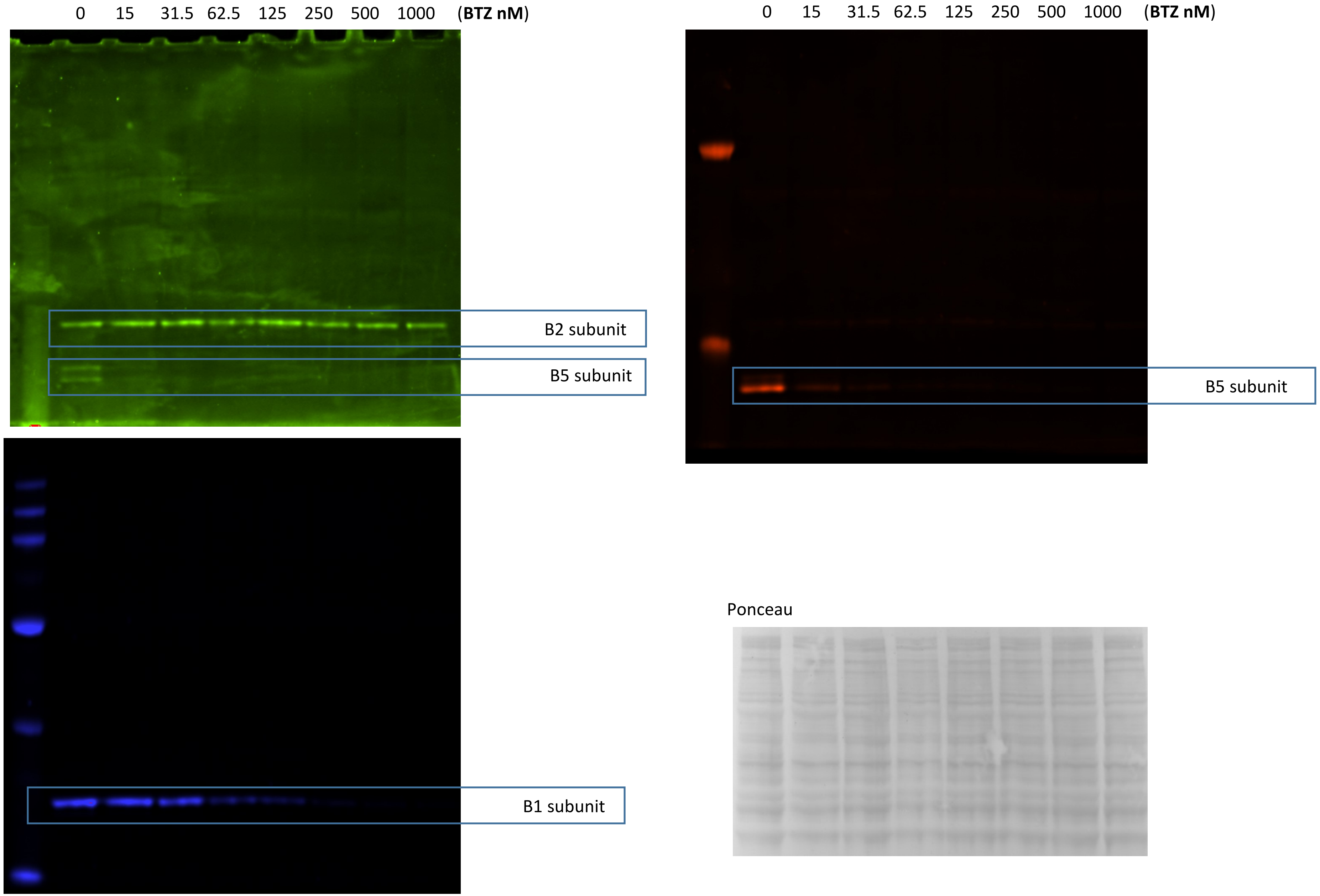
0 15 31.5 62.5 125 250 500 1000 (BTZ nM)
B2 subunit
B5 subunit
B1 subunit
0 15 31.5 62.5 125 250 500 1000 (BTZ nM)
B5 subunit
Ponceau

H157

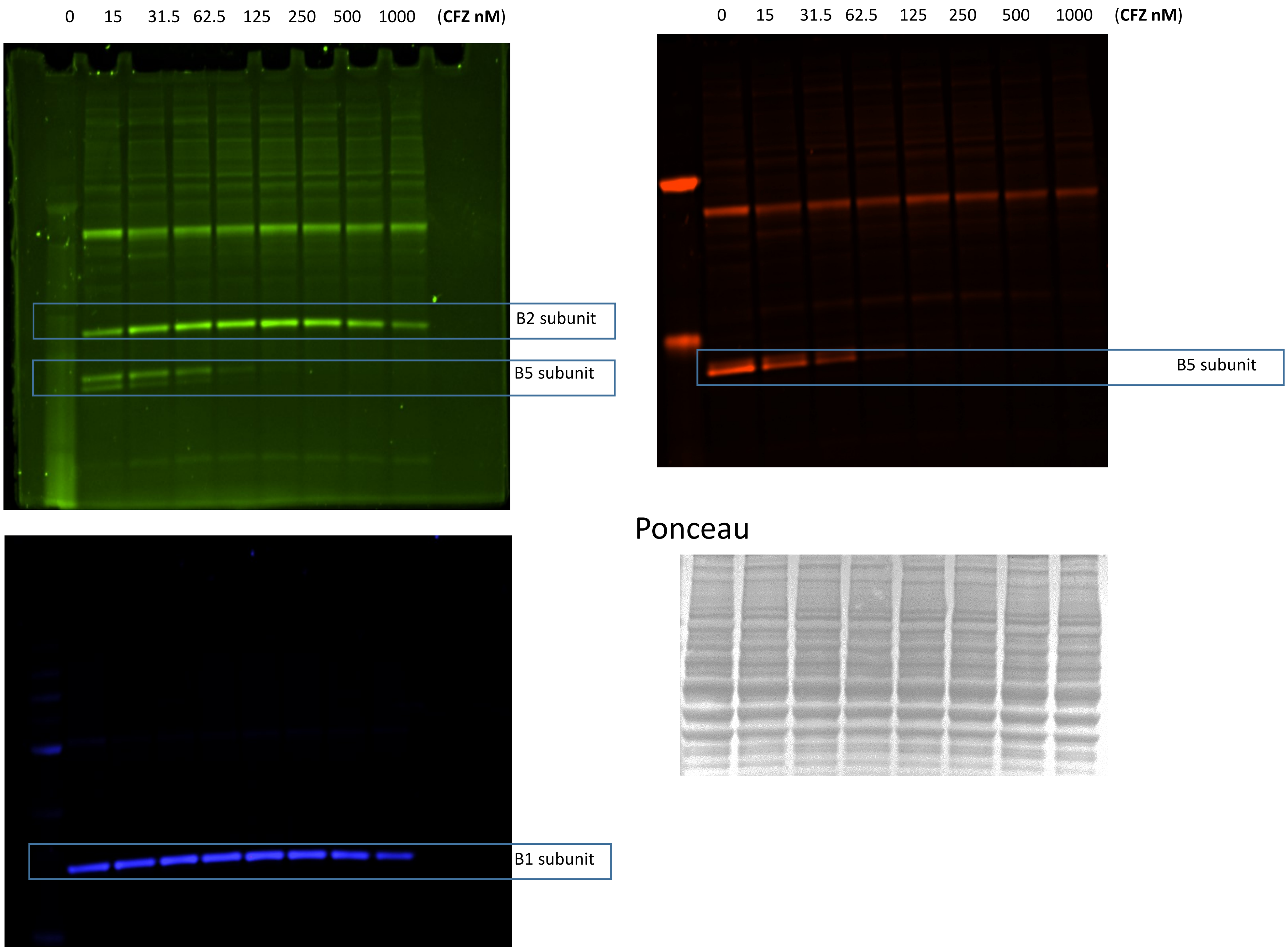
0 15 31.5 62.5 125 250 500 1000 (CFZ nM)
B2 subunit
B5 subunit
0 15 31.5 62.5 125 250 500 1000 (CFZ nM)
B5 subunit
Ponceau
B1 subunit

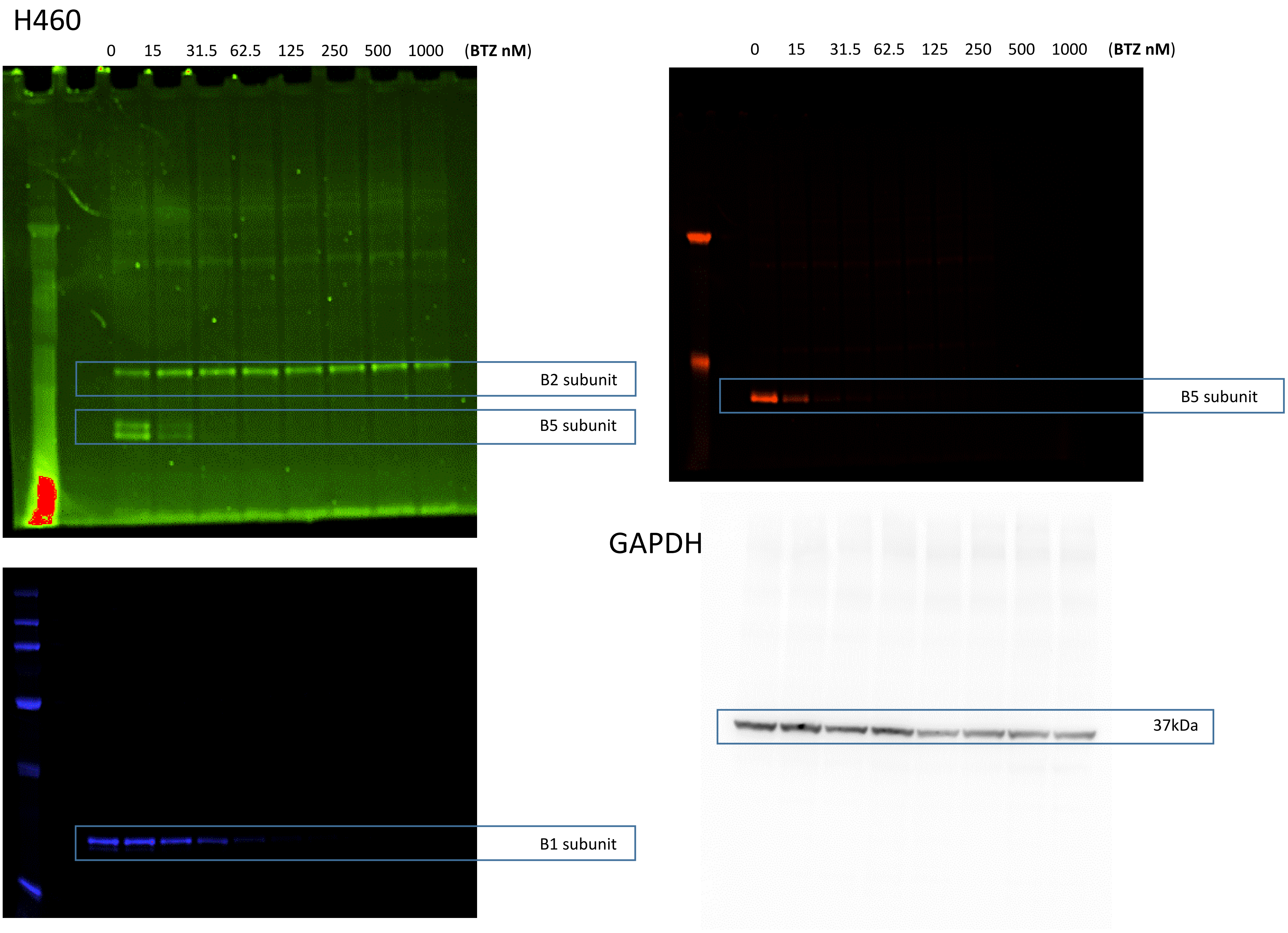
H460
0 15 31.5 62.5 125 250 500 1000 (BTZ nM)
B2 subunit
B5 subunit
B1 subunit
0 15 31.5 62.5 125 250 500 1000 (BTZ nM)
B5 subunit
GAPDH
37kDa

H460

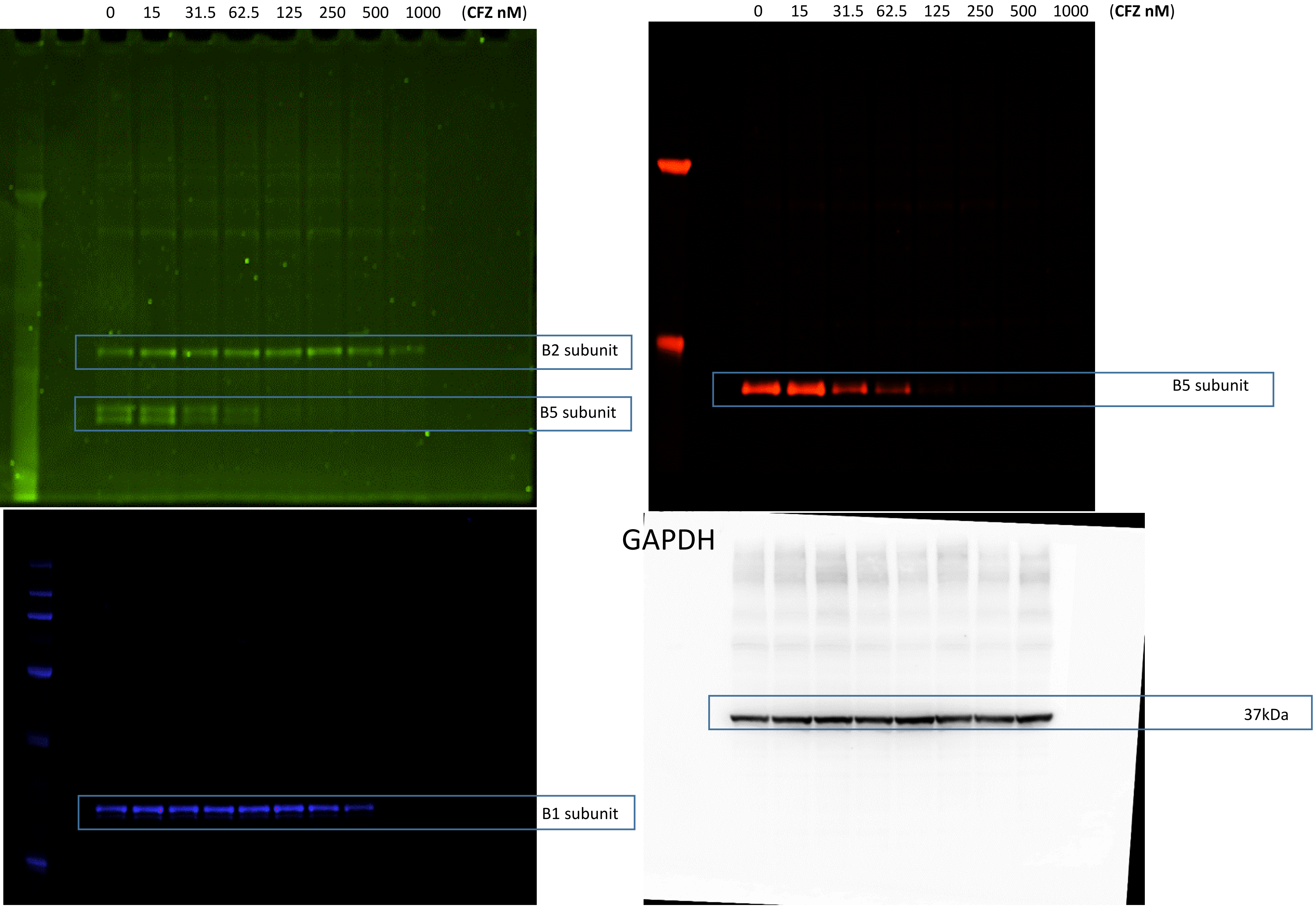
0 15 31.5 62.5 125 250 500 1000 (CFZ nM)
B2 subunit
B5 subunit
0 15 31.5 62.5 125 250 500 1000 (CFZ nM)
B5 subunit
B1 subunit
GAPDH
37kDa

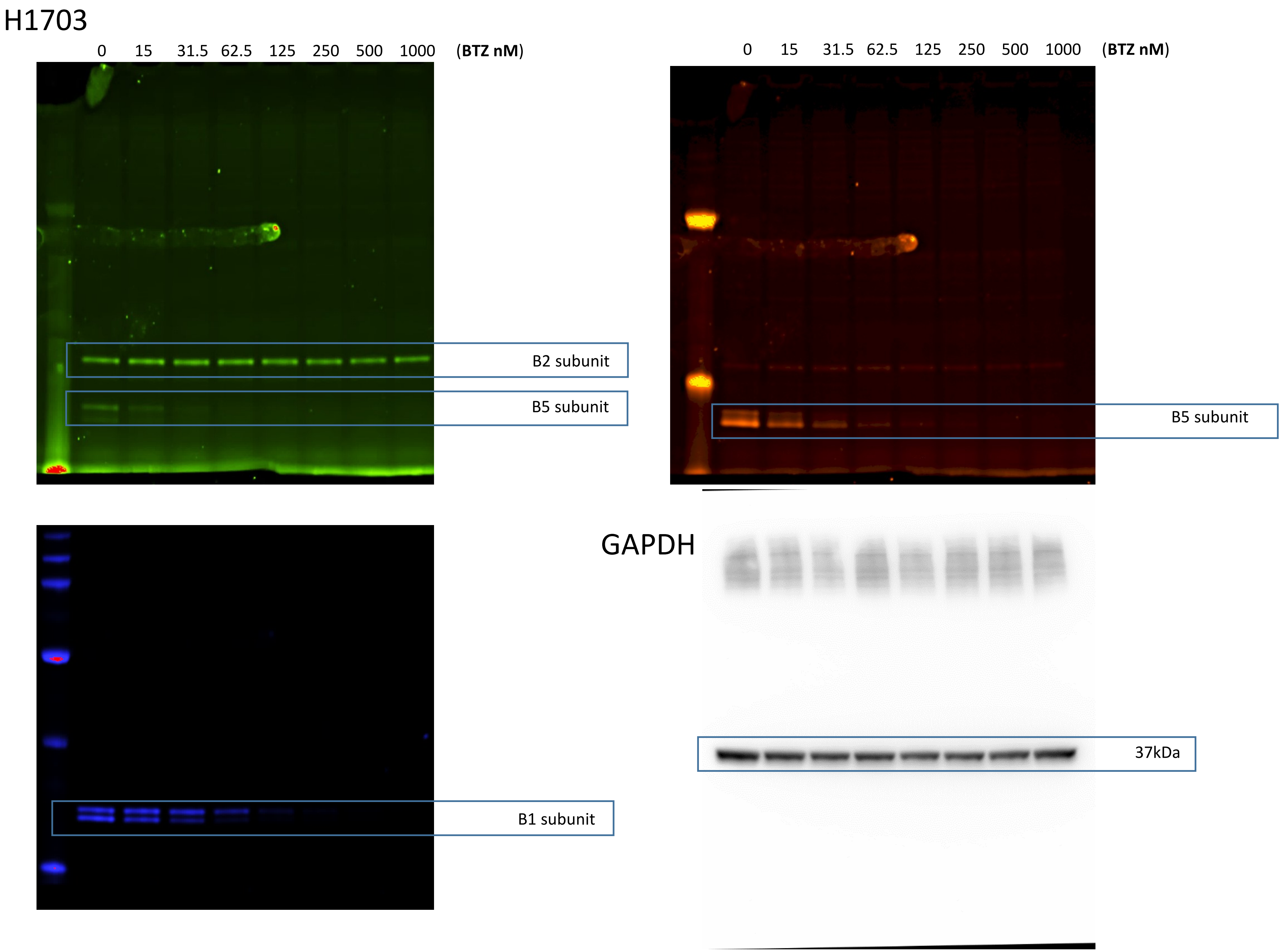
H1703
0 15 31.5 62.5 125 250 500 1000 (BTZ nM)
B2 subunit
B5 subunit
0 15 31.5 62.5 125 250 500 1000 (BTZ nM)
B5 subunit
B1 subunit
GAPDH
37kDa

H1703

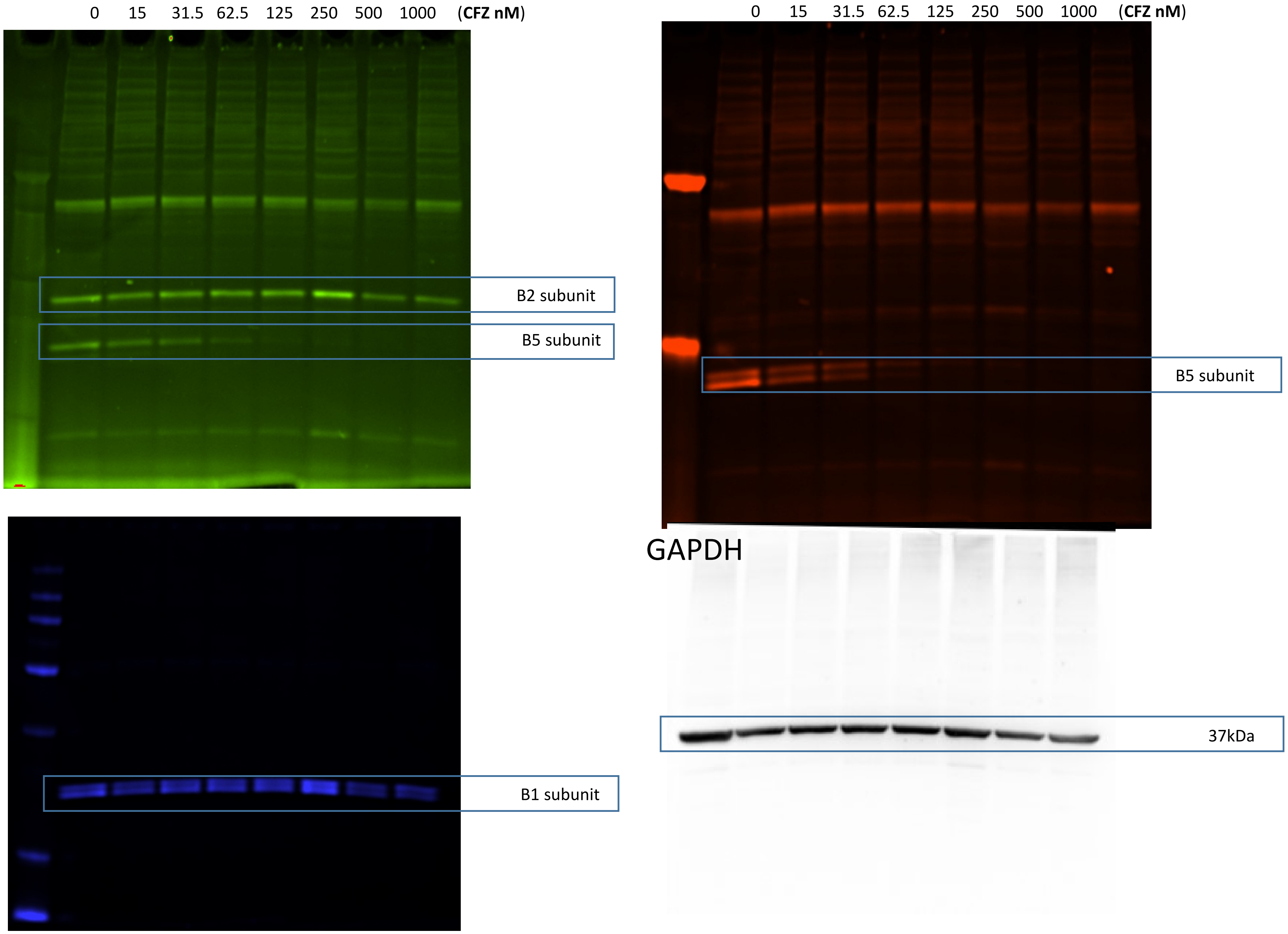
0 15 31.5 62.5 125 250 500 1000 (CFZ nM)
B2 subunit
B5 subunit
0 15 31.5 62.5 125 250 500 1000 (CFZ nM)
B5 subunit
GAPDH
37kDa
B1 subunit

Originals for Fig S3

A549

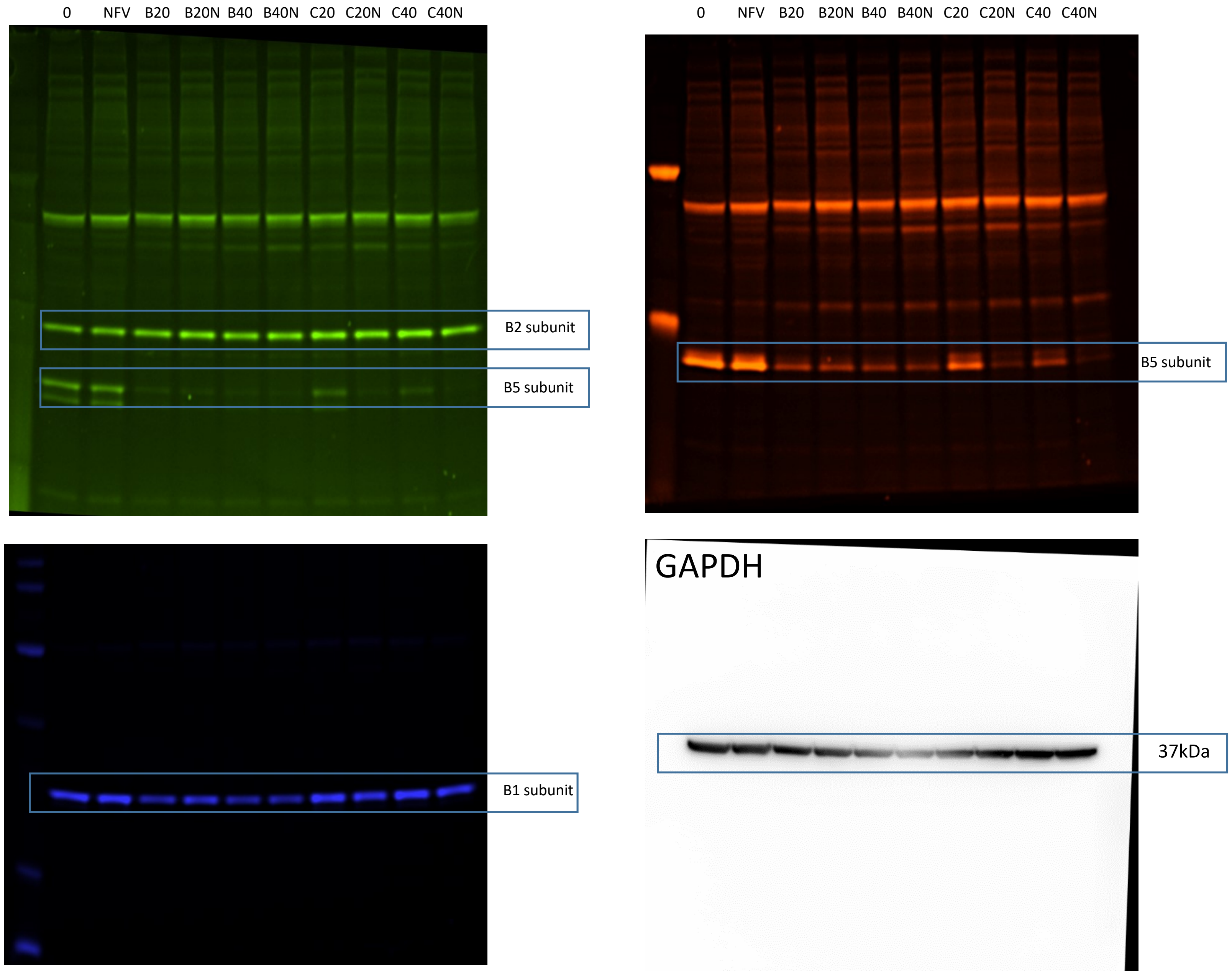

0 NFV B20 B20N B40 B40N C20 C20N C40 C40N
0 NFV B20 B20N B40 B40N C20 C20N C40 C40N
B2 subunit
B5 subunit
B5 subunit
B1 subunit
GAPDH
37kDa

H157

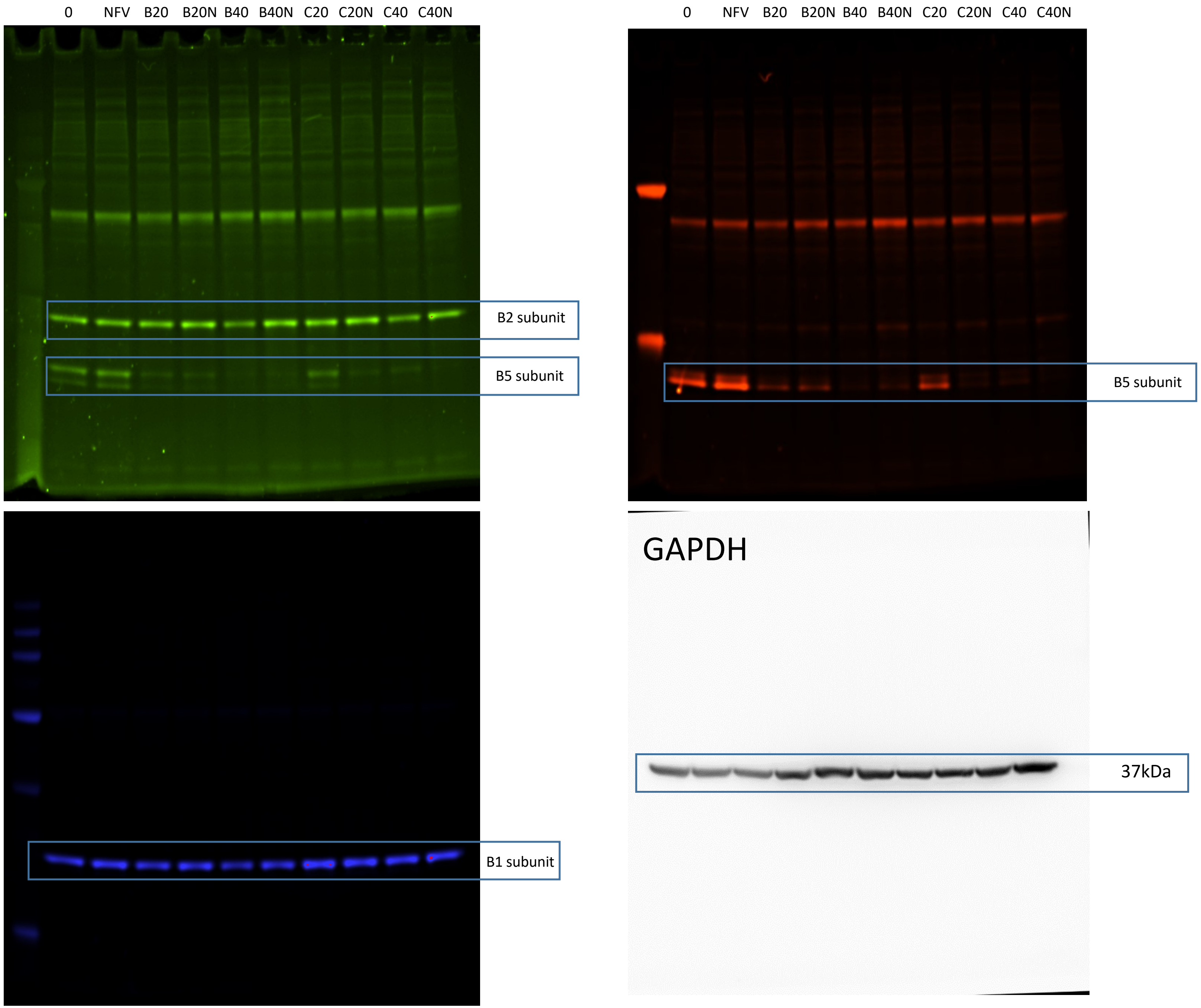
0
NFV
B20
B20N
B40
B40N
C20
C20N
C40
C40N
B2 subunit
B5 subunit
0
NFV
B20
B20N
B40
B40N
C20
C20N
C40
C40N
B5 subunit
B1 subunit
GAPDH
37kDa

H460

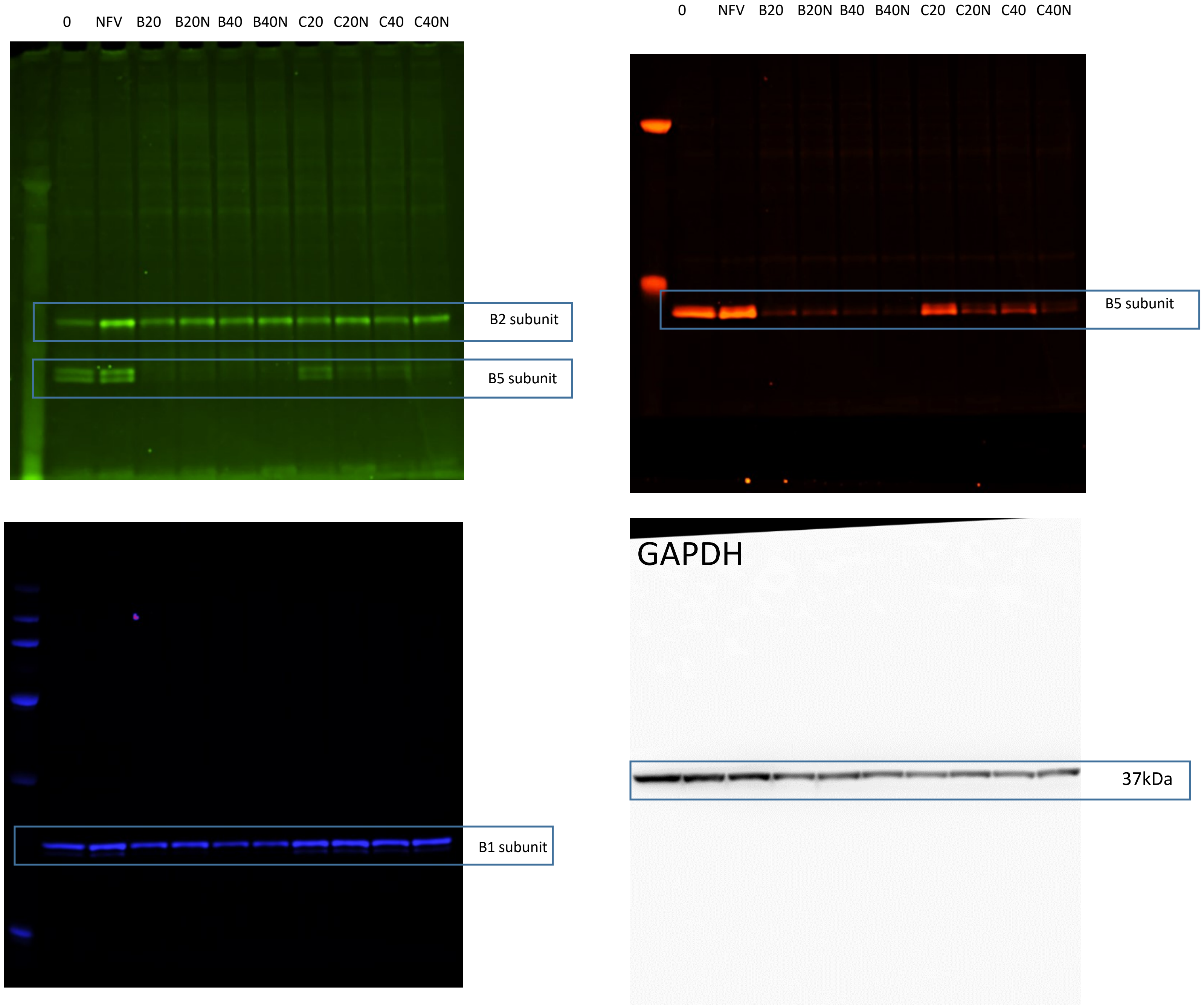
0 NFV B20 B20N B40 B40N C20 C20N C40 C40N
B2 subunit
B5 subunit
0 NFV B20 B20N B40 B40N C20 C20N C40 C40N
B5 subunit
B1 subunit
GAPDH
37kDa

H1703

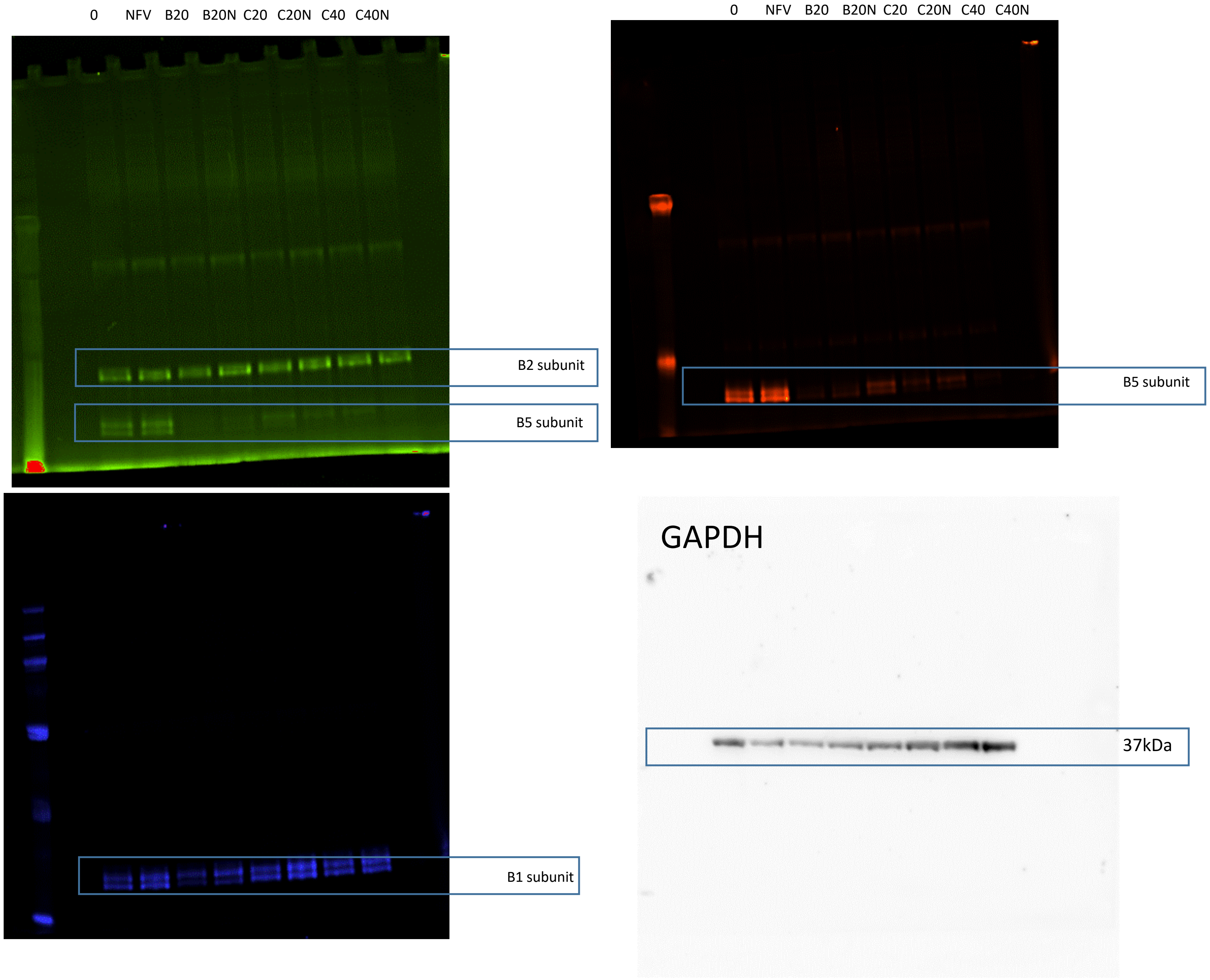
0 NFV B20 B20N C20 C20N C40 C40N
B2 subunit
B5 subunit
0 NFV B20 B20N C20 C20N C40 C40N
B5 subunit
B1 subunit
GAPDH
37kDa
