# Supplementary material for: The cytotoxic activity of carfilzomib together with nelfinavir is superior to the bortezomib/nelfinavir combination in non-small cell lung carcinoma
Source: Sci Rep. 2023 Mar 17;13:4411. doi: 10.1038/s41598-023-31400-6 (PMC10023769; doi:10.1038/s41598-023-31400-6)

The cytotoxic activity of carfilzomib together with nelfinavir is superior to the bortezomib/nelfinavir combination in non-small cell lung carcinoma

Lenka Besse, Marianne Kraus, Andrej Besse, Christoph Driessen, Ignazio Tarantino

Raw images of Western blot membranes

Originals for Fig. 3A

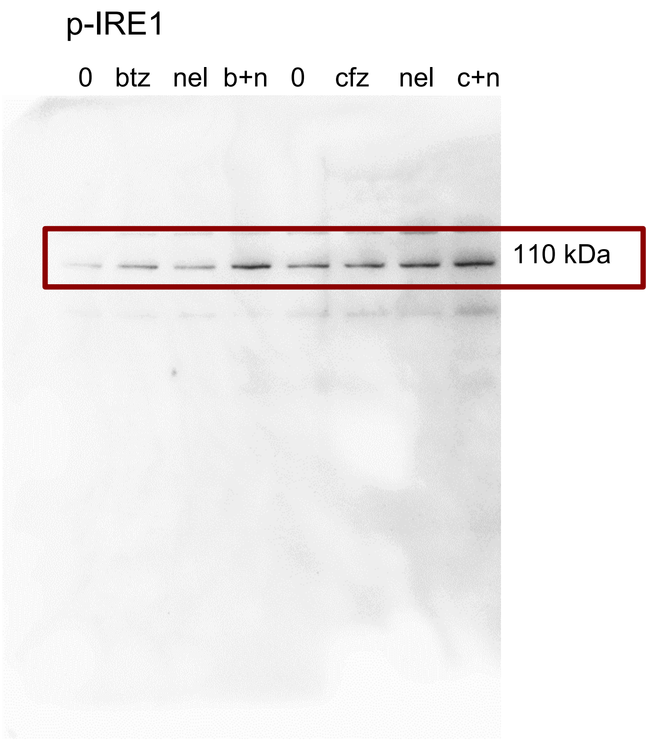

ATF6

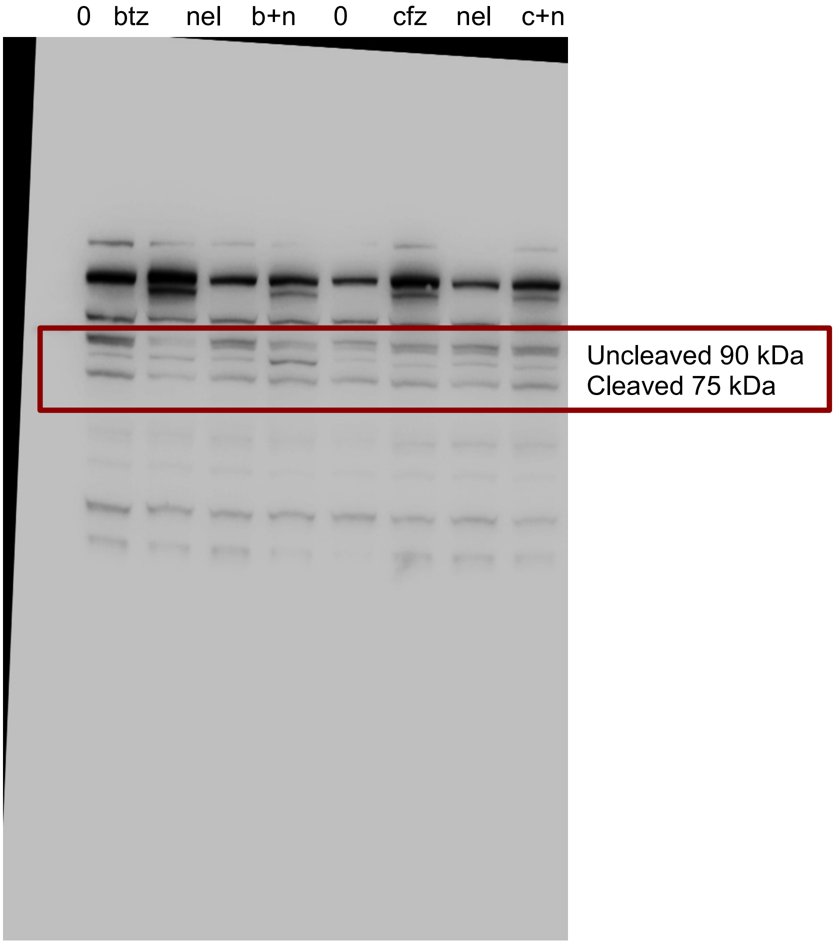
0 btz nel b+n 0 cfz nel c+n
Uncleaved 90 kDa
Cleaved 75 kDa

ATF4

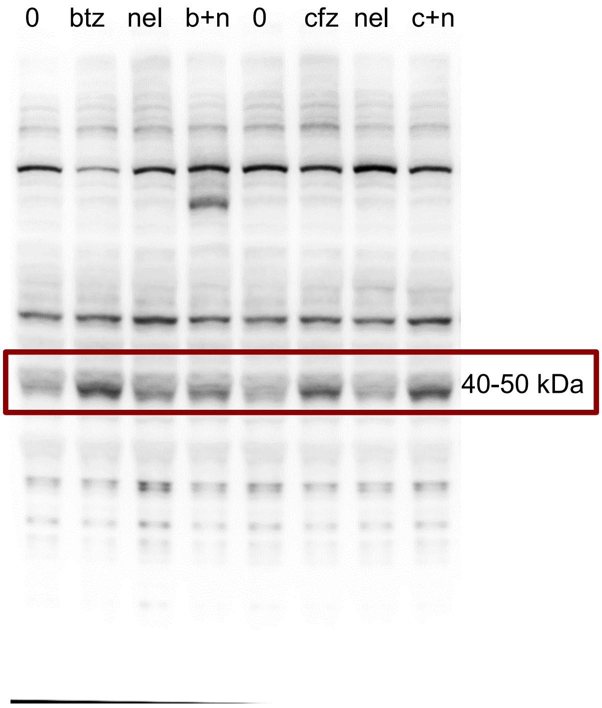
0 btz nel b+n 0 cfz nel c+n
40-50 kDa

BIP

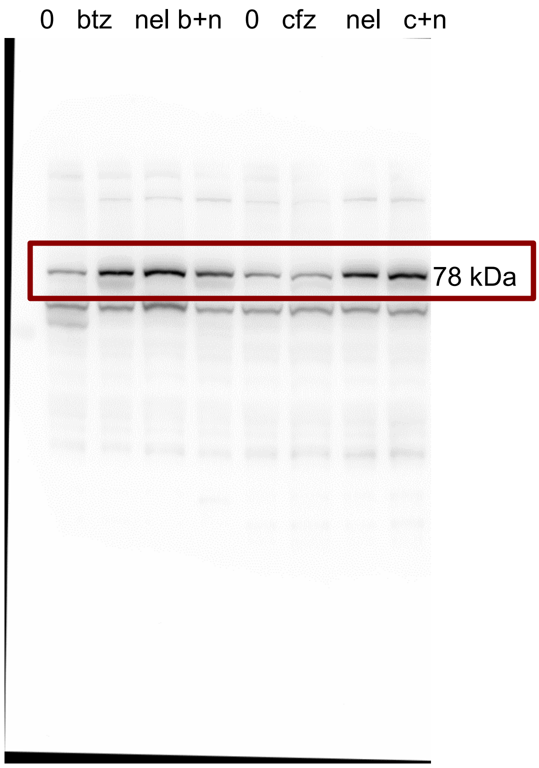

PDI

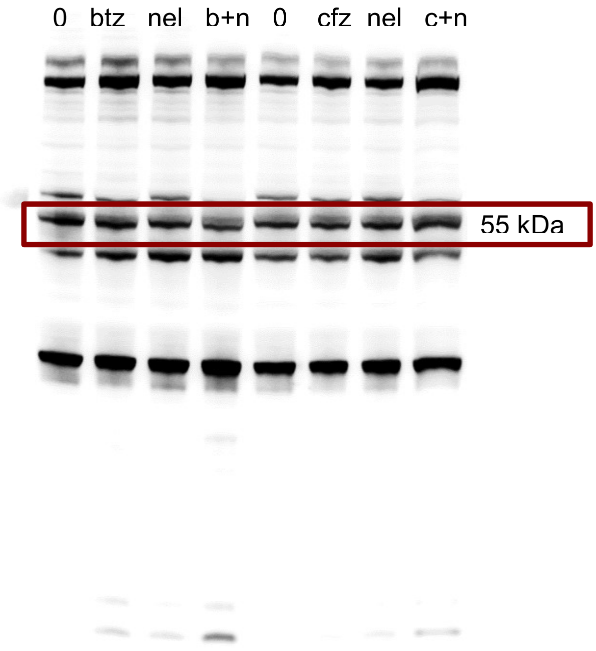

CHOP

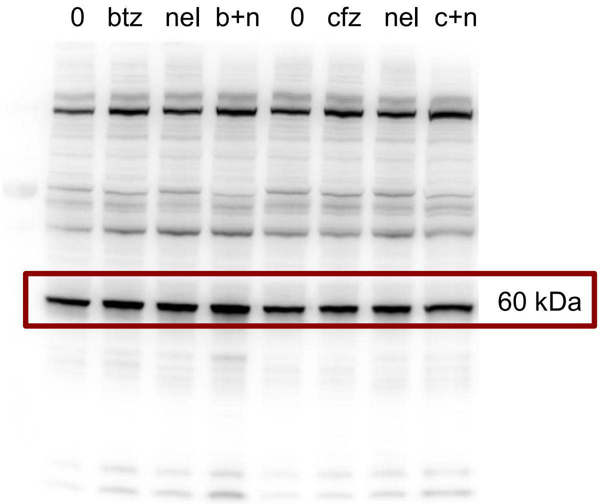

GAPDH

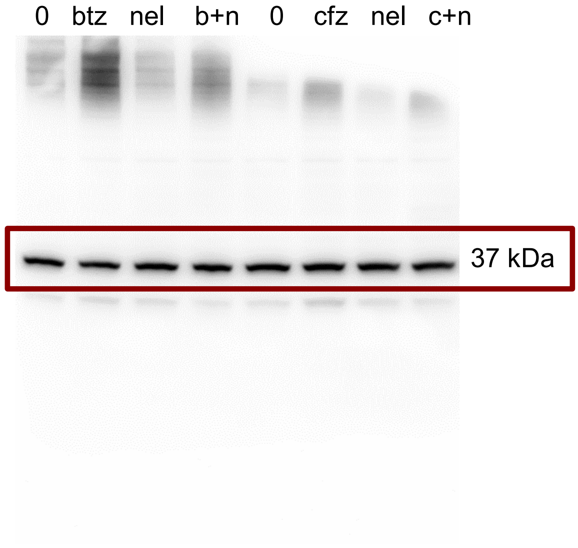

Originals for Fig. 3C

Poly-Ub

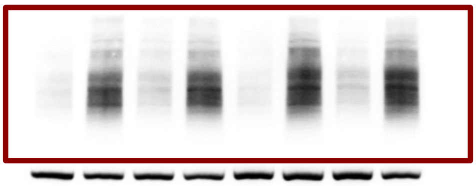

GAPDH

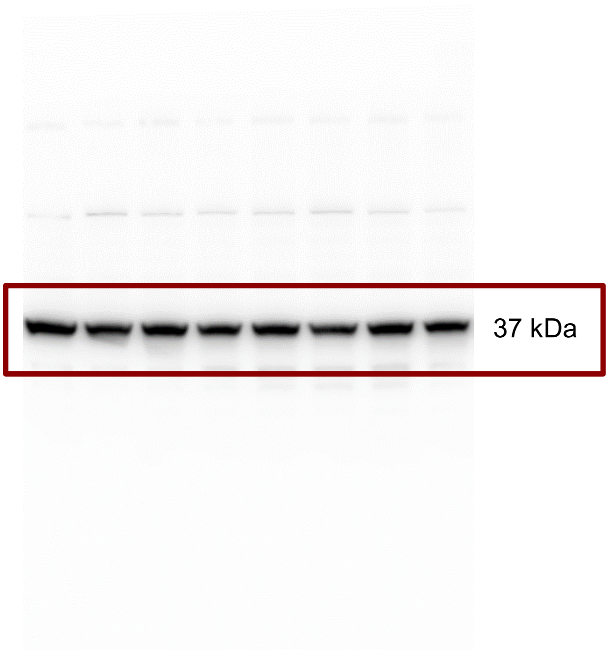

Originals for Fig. 3D

MCL-1

0 btz nel b+n 0 cfz nel c+n

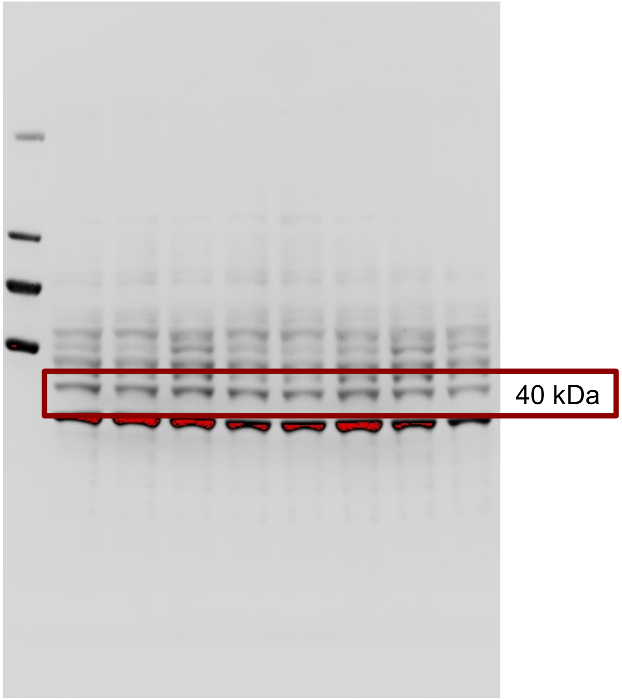

NOXA

0 btz nel b+n 0 cfz nel c+n

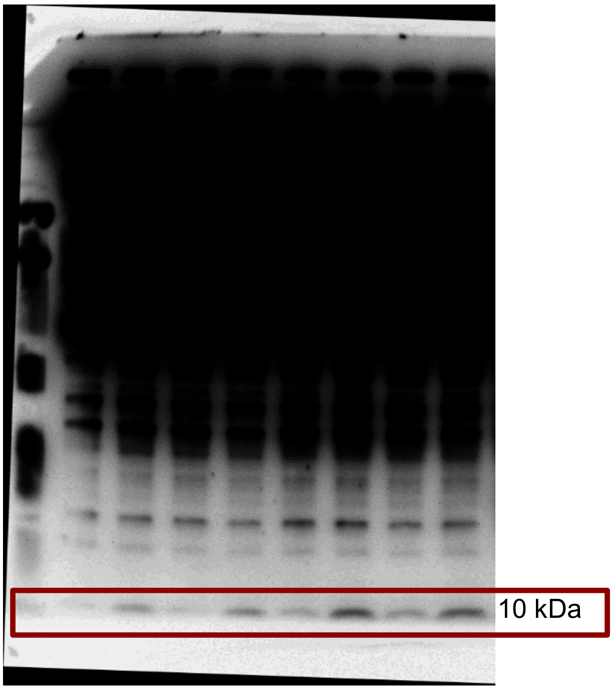

BIM

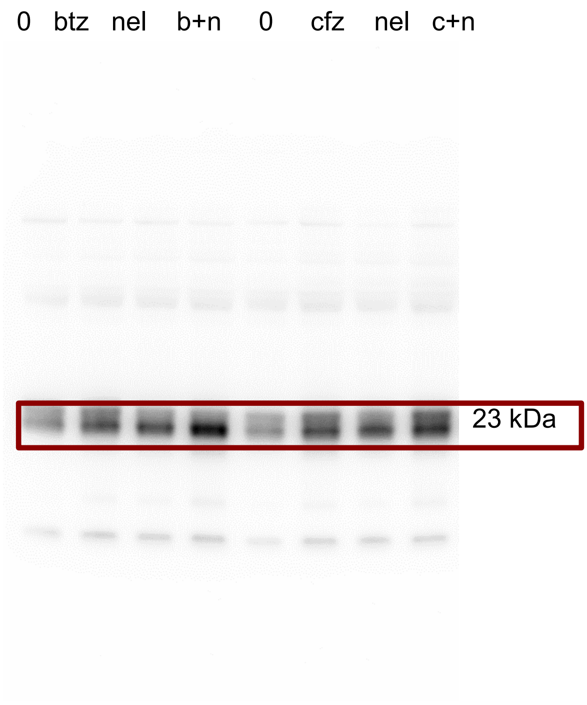

Caspase 3 cleaved

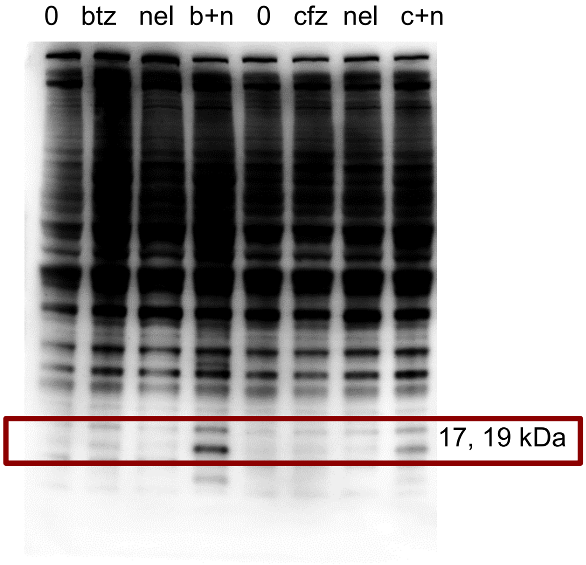

Caspase 7 cleaved

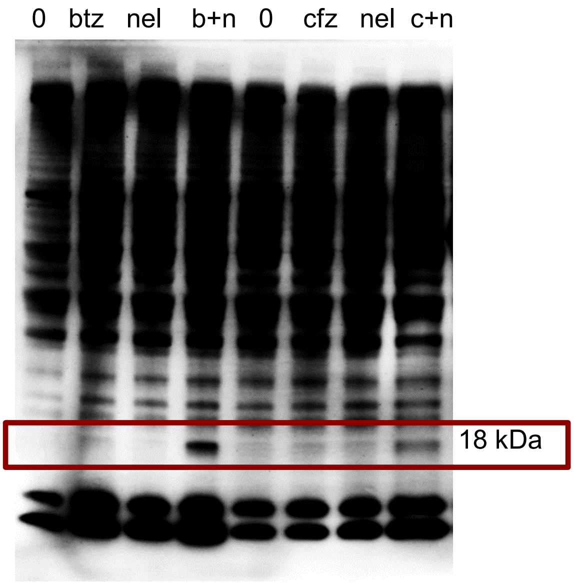

Caspase 9 cleaved

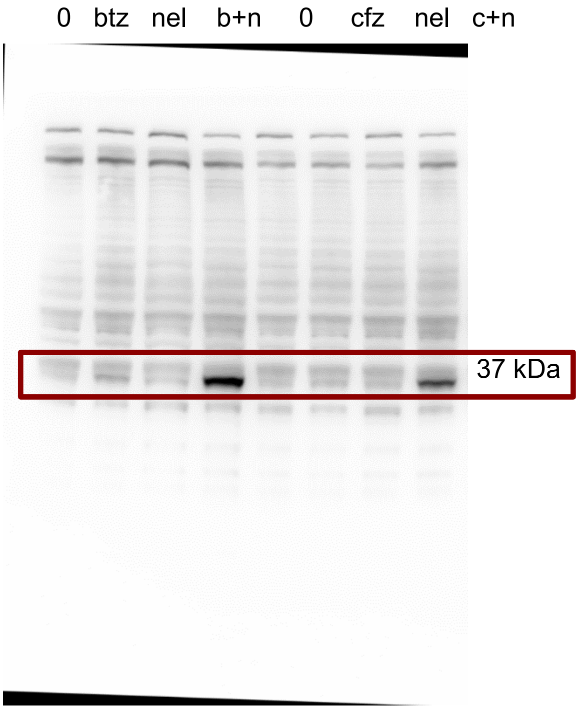

GAPDH

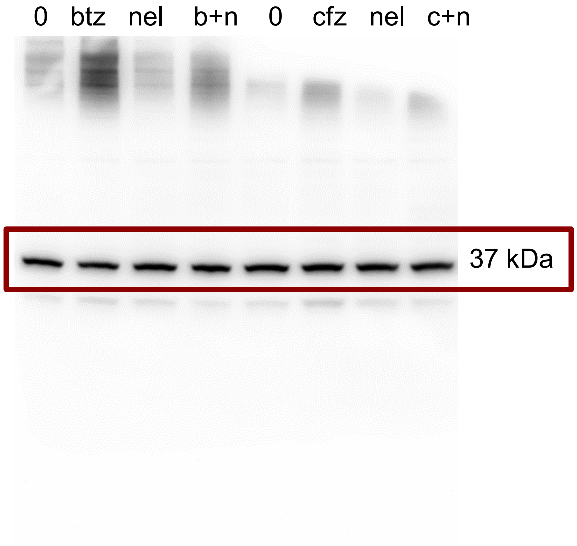

LC3B

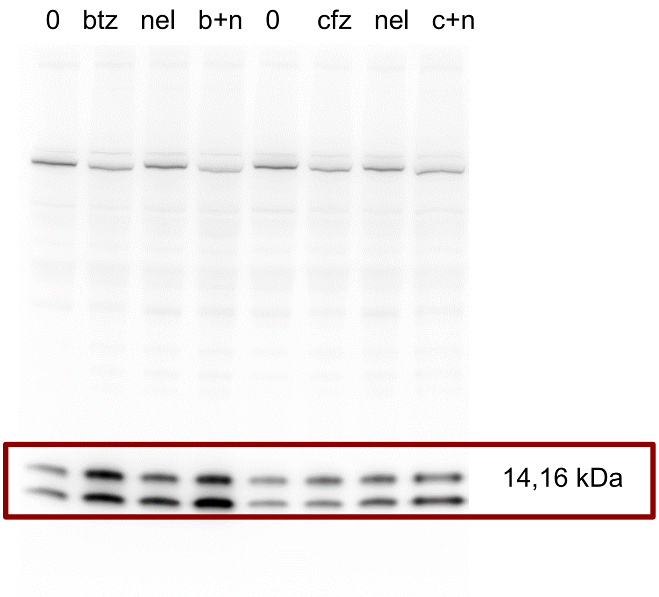

GAPDH

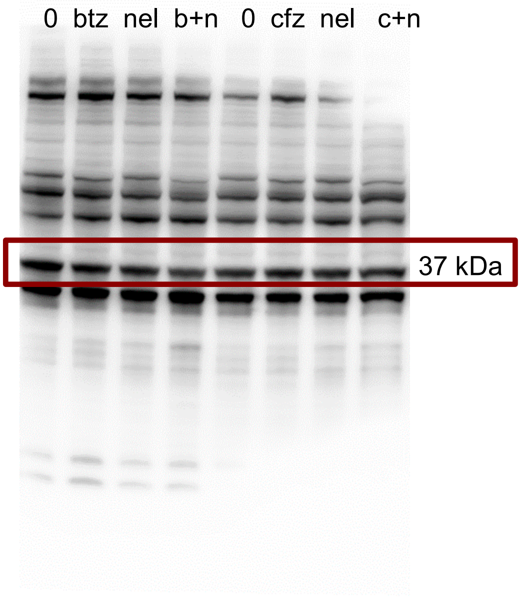

Originals for Fig. 5B

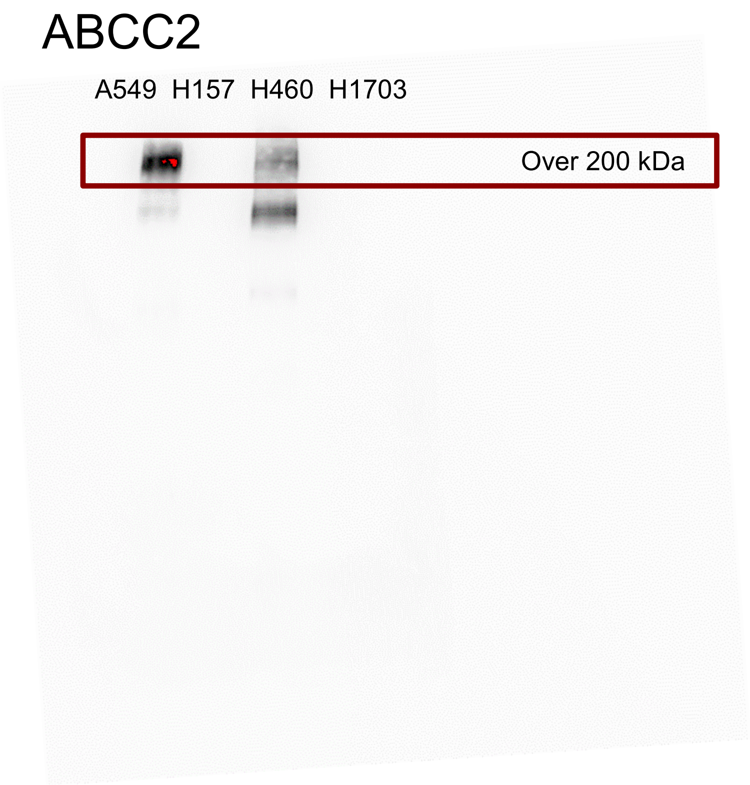

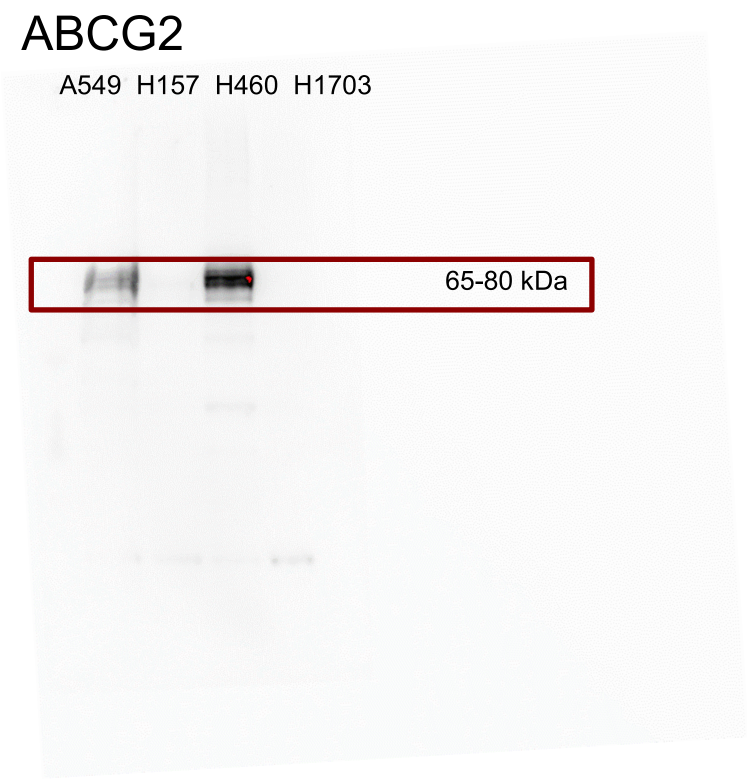

GAPDH

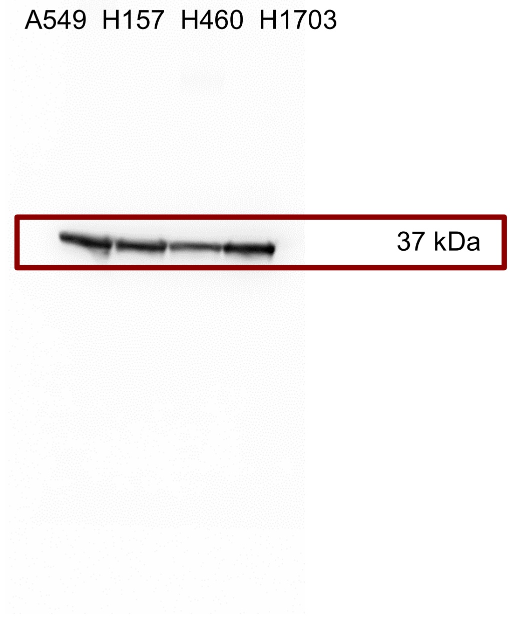

Supplement: Supplementary file 3 — Supplementary Information 3. [file 41598_2023_31400_MOESM3_ESM.pdf]
